# PFOS-induced mitochondrial ROS impairs male reproductive function by suppressing FTO-dependent m^6^A RNA demethylation

**DOI:** 10.1016/j.jbc.2026.113488

**Published:** 2026-08-26

**Authors:** Yipeng Pang, Shiqing Yang, Yuenan Wang, Fructueux Modeste Amona, Rong Zhou, Zilu Liu, Chuanbei Tian, Xi Chen

**Affiliations:** Institute of Cellular and Molecular Biology, School of Life Science, Jiangsu Normal University, Xuzhou, Jiangsu, China

**Keywords:** m^6^A dysregulation, male reproductive dysfunction, mitochondrial ROS, PFOS, FTO

## Abstract

Perfluorooctanesulfonic acid (PFOS), a persistent per- and polyfluoroalkyl substance, has been associated with male reproductive toxicity, but the underlying molecular mechanisms remain incompletely defined. Here, we show that chronic exposure to environmentally relevant doses of PFOS caused dose-dependent testicular injury and spermatogenic dysfunction in mice, as indicated by reduced testis weight, disrupted seminiferous tubule architecture, and impaired sperm quality. Transcriptomic profiling revealed marked enrichment of pathways related to oxidative stress, mitochondrial dysfunction, and reproductive regulation. Mechanistically, PFOS increased mitochondrial reactive oxygen species (ROS) production in GC-2 spermatocyte cells, leading to mitochondrial membrane potential collapse, lipid peroxidation, and depletion of antioxidant defenses. Pharmacological scavenging of mitochondrial ROS with Mito-TEMPO attenuated PFOS-induced mitochondrial injury *in vitro* and improved testicular damage and sperm quality *in vivo*. We further found that PFOS suppressed the RNA demethylase fat mass and obesity-associated protein (FTO) and promoted its ROS-dependent redistribution from the nucleus to mitochondria, resulting in global N6-methyladenosine (m^6^A) hypermethylation. Integrated m^6^A epitranscriptomic analysis showed that PFOS remodeled m^6^A modification patterns on key transcripts involved in spermatogenesis, steroidogenesis, and redox homeostasis, including CYP11A1, SOX9, STRA8, and glutathione peroxidase (GPX4). FTO bound these transcripts, and restoration of FTO expression reversed PFOS-induced m^6^A hypermethylation and rescued reproductive gene expression. Together, these findings identify a mitochondrial ROS–FTO–m^6^A regulatory axis that links PFOS-induced mitochondrial oxidative stress to epitranscriptomic disruption and male reproductive dysfunction. Our study defines FTO-dependent RNA demethylation as a critical molecular mechanism and potential intervention target in PFOS-induced reproductive injury.

Chronic exposure to hazardous environmental chemicals has become a major global health concern, with increasing evidence linking environmental pollution to declining human reproductive capacity (1, 2). Epidemiological studies report a continuous decrease in global fertility rates, accompanied by an annual decline of approximately 2 to 3% in sperm concentration, trends closely associated with exposure to environmental contaminants (3). Among these, Perfluorooctanesulfonic acid (PFOS), a representative per- and polyfluoroalkyl (PFAS), is of particular concern due to its exceptional environmental persistence, bioaccumulation, and global distribution (4, 5). PFOS's carbon–fluorine bonds make it resistant to hydrolysis, photolysis, and biodegradation (6), facilitating its continuous release from industrial and consumer products and its widespread dissemination through environmental and food-chain pathways (7). Consequently, PFOS accumulates in human tissues and poses long-term health risks (8, 9). Both epidemiological and experimental studies have linked PFOS to reduced sperm quality, abnormal morphology, and impaired spermatogenesis (8). PFOS disrupts testicular function through pathways such as suppressing antioxidant systems, causing excessive mitochondrial ROS production and structural damage to seminiferous tubules (10), activating apoptosis and autophagy in germ cells, disrupting sertoli cell junctions, and impairing leydig cell steroidogenesis (11). These effects reduce sperm quality and can be inherited, with gestational exposure decreasing sperm counts, testosterone, and steroidogenic gene expression in offspring (12). Despite complex mechanisms, these effects mainly stem from mitochondrial dysfunction.

Mitochondrial dysfunction is a key driver of oxidative stress (13). As the primary intracellular source of ROS, mitochondria regulate both energy metabolism and redox homeostasis (14). PFOS exposure disrupts mitochondrial membrane potential and electron transport chain function, resulting in excessive ROS generation (15). When ROS production, which exceeds antioxidant capacity, and oxidative stress is established and progressively amplified. Mitochondria-derived ROS initially damage respiratory chain components, creating a self-reinforcing cycle of mitochondrial impairment and ROS overproduction (16, 17). Subsequently, ROS propagate to the cytoplasm and nucleus, inducing lipid peroxidation, protein oxidation, and DNA damage, while activating redox-sensitive signaling pathways such as p38 MAPK and NF-κB (18, 19, 20). Beyond these classical effects, oxidative stress has emerged as an important regulator of epitranscriptomic modifications (21).

N6-methyladenosine (m^6^A), the most abundant internal modification in eukaryotic mRNA, is dynamically regulated by methyltransferases, demethylases, and reader proteins, thereby controlling mRNA stability, splicing, and translation (22, 23). Increasing evidence indicates that oxidative stress and m^6^A modification are functionally interconnected. ROS can regulate the expression and activity of m^6^A-related enzymes *via* signaling pathways such as NF-κB and HIF-1α, whereas m^6^A modification modulates mitochondrial function by controlling transcripts involved in oxidative phosphorylation and mitochondrial dynamics (24, 25, 26). This bidirectional interaction suggests the existence of an integrated “oxidative stress–epitranscriptomic” regulatory axis. While this axis has been implicated in several pathological conditions (24, 25), its role in environmental toxicology, particularly in male reproduction, remains largely unexplored. Given the dynamic mitochondrial remodeling and chromatin reorganization during spermatogenesis, germ cells may be especially vulnerable to environmentally induced epitranscriptomic dysregulation (27). Although PFAS exposure has been associated with altered DNA methylation in sperm (28), the role of m^6^A RNA modification, which represents a more dynamic and reversible layer of gene regulation, remains unclear in PFOS-induced reproductive toxicity.

Herein, this study investigates how the mitochondrial ROS–m^6^A regulatory network contributes to PFOS-induced male reproductive injury. It hypothesizes that PFOS disrupts mitochondrial redox balance, leading to excess ROS that modulates FTO and affects m^6^A regulation. This may lead to abnormal gene methylation involved in spermatogenesis and steroidogenesis, contributing to testicular dysfunction and poor sperm development. The study also explores targeting mitochondrial ROS to reduce these effects. Understanding the mitochondrial ROS–FTO/m^6^A axis could reveal how environmental contaminants harm male reproductive health and guide risk mitigation for PFAS exposure.

## Results

### Environmental PFOS exposure induces testicular injury and spermatogenic dysfunction in mice

To evaluate the long-term reproductive toxicity of PFOS, male ICR mice were given moderate, environmentally relevant doses of PFOS (0.1, 0.5, and 5 mg/kg/day) in drinking water for 8 weeks (Fig. 1*A*). There were no noticeable differences in overall appearance among the groups. However, testicular size progressively decreased as PFOS doses increased, with the most severe testicular atrophy observed in the 5 mg/kg/day group (Fig. 1*B*). Although gross changes in testicular size were relatively subtle, quantitative analysis demonstrated a dose-dependent reduction in the testis-to-body weight ratio following PFOS exposure (Fig. 1*F*). Spermatogenic impairment further confirmed alterations in the sperm parameters. PFOS exposure at 0.1 mg/kg/day did not significantly affect sperm count, whereas exposure to 0.5 and 5 mg/kg/day significantly reduced sperm number (Fig. 1*C*). Similar patterns were observed for sperm morphology showing no significant change at 0.1 mg/kg/day, but notable increases in sperm abnormalities at 0.5 and 5 mg/kg/day, especially at the highest dose (Fig. 1*D*). These findings demonstrate that long-term PFOS exposure adversely affects both sperm production and sperm quality.

**Figure 1:**
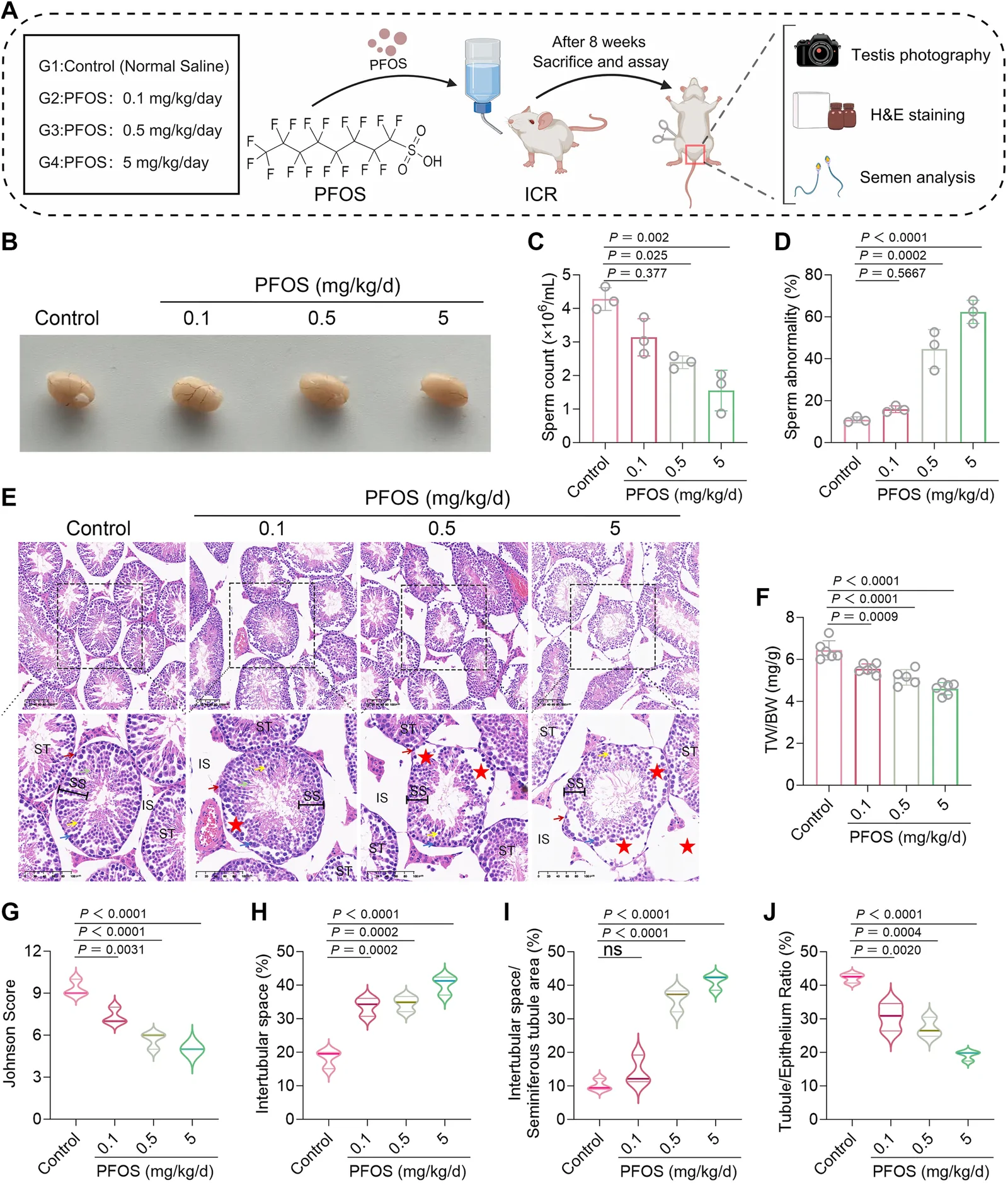
**Chronic PFOS exposure induces dose-dependent spermatogenic dysfunction and testicular structural damage in mice.***A,* schematic illustration of the experimental design for PFOS exposure in ICR male mice. Mice were exposed to PFOS *via* drinking water for 8 weeks *B,* gross testes morphology from control mice and mice exposed to PFOS at 0.1, 0.5, and 5 mg/kg/day. *C,* quantification of epididymal sperm counts following PFOS exposure. *D,* quantification of abnormal sperm morphology percentages. *E,* H&E–stained sections of testicular tissue. *Red asterisks* indicate degenerated seminiferous tubules and disrupted spermatogenic epithelium. The scale bar represents 100 μm. *F,* quantification of testis-to-body weight ratio. *G,* johnsen score evaluating spermatogenic maturation. *H,* quantification of intertubular space area. *I,* intertubular space-to-seminiferous tubule area. *J,* tubule-to-epithelium ratio. Data are presented as mean ± SD (n = 6 per group). PFOS, perfluorooctanesulfonic acid.

Histopathology demonstrated dose-dependent testicular injury, with well-organized seminiferous tubules, intact spermatogenic epithelium, and abundant sperm. In contrast, the 0.1 mg/kg/day group showed mild thinning of the seminiferous epithelium and vacuolation. The 0.5 mg/kg/day group showed seminiferous tubular atrophy, increased germ cell exfoliation, reduced sperm count, and interstitial edema. The 5 mg/kg/day group had severe seminiferous tubule disruption, extensive epithelial loss, almost no sperm, and inflammation (Fig. 1*E*). Morphometric analysis aligned with histological findings, showing Johnsen scores (29) significantly decreased (Fig. 1*G*) and interstitial space increased (Fig. 1*H*) in all PFOS-exposed groups, while the relative intertubular space increased (Fig. 1*I*) and the tubule-to-epithelium ratio decreased (Fig. 1*J*). Together, these results demonstrated that long-term PFOS exposure, even at environmentally relevant doses, can induce dose-dependent testicular atrophy, seminiferous tubule disruption, and impaired spermatogenesis in mice.

### PFOS induces testicular transcriptomic remodeling toward oxidative stress and mitochondrial dysfunction

Recently, PFOS exposure has been shown to dysregulate cell-specific transcription in the mouse spermatogenic microenvironment (30), but the mechanisms by which oxidative stress and mitochondrial dysfunction are significantly involved remain unclear. To gain more insight into PFOS-induced reproductive toxicity mechanisms, transcriptomic analysis was first performed on testicular tissues from control and PFOS-exposed mice (5 mg/kg/d). A total of 376 differentially expressed genes (DEGs) were identified, with 192 upregulated and 184 downregulated compared with the control group (Fig. 2*A*), indicating testicular transcriptome reprogramming. Gene Ontology (GO) enrichment analysis revealed that DEGs are linked to biological processes such as oxidative stress, metabolic regulation, and reproduction (Fig. 2*B*). Notably, terms related to spermatogenesis, sperm differentiation, mitochondrial function, and response to oxidative stress were significantly enriched (Fig. 2*C*), suggesting that PFOS exposure disrupts both redox homeostasis and reproductive processes. DEGs were also enriched in catalytic activity, antioxidant activity, and transcriptional regulation, supporting the involvement of enzymatic and redox-related processes.

**Figure 2:**
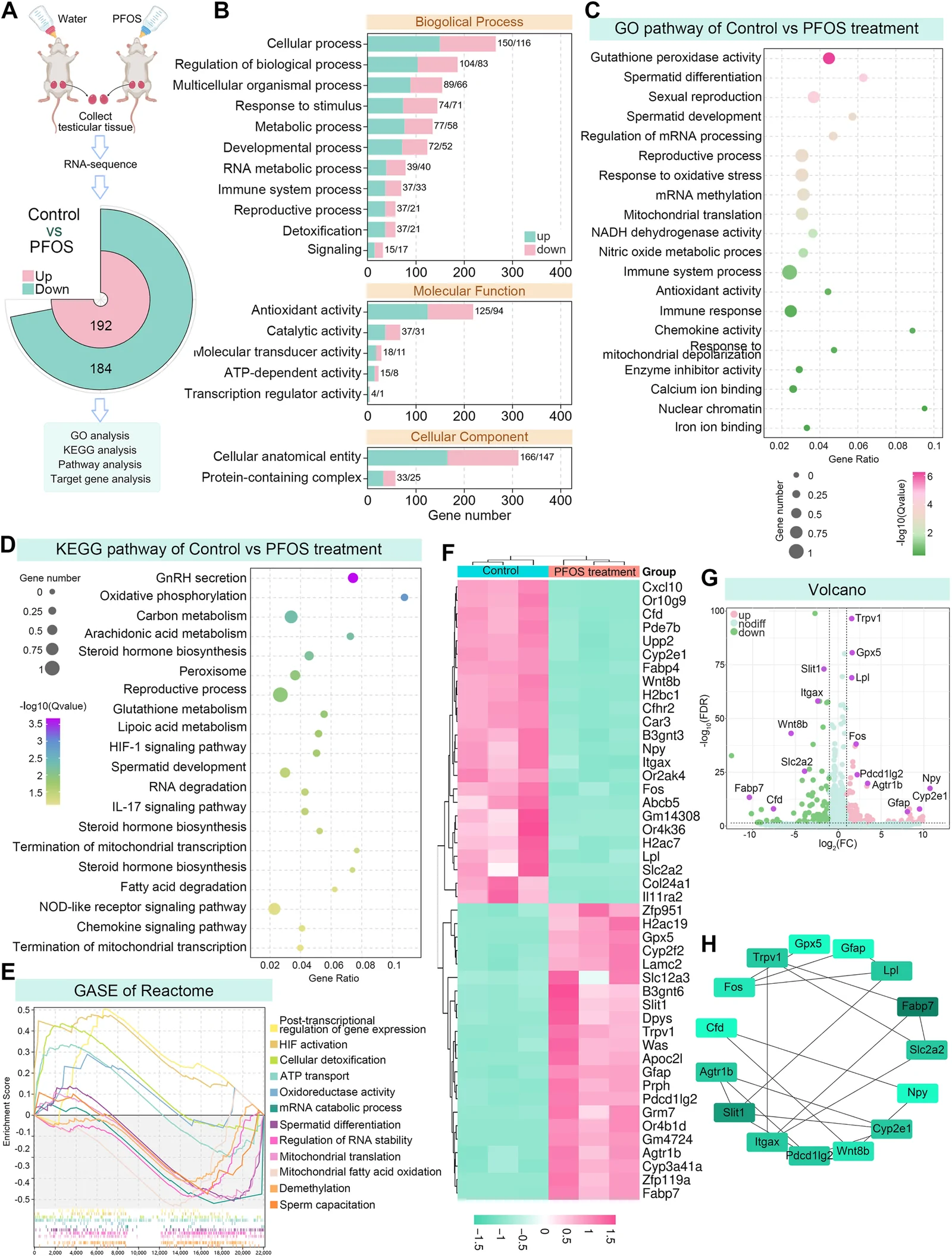
**PFOS exposure induces testicular transcriptome remodeling enriched in oxidative stress, mitochondrial function, and spermatogenesis pathways.***A,* schematic workflow and pie chart showing the number of changed genes (upregulated and downregulated) in PFOS-exposed testes mice compared to controls. *B,* GO analysis of DEGs, and *(C)* key pathways analysis of the PFOS exposure effects. *D,* KEGG pathway enrichment analysis of DEGs affected by PFOS. *E,* Reactome Gene Set Enrichment Analysis of DEGs, showing enrichment scores for pathways related to posttranscriptional gene regulation. *F,* heat map of targeted genes from enriched pathways, with focus on key regulators of oxidative stress and spermatogenesis. *G, volcano plot* analysis of DEGs in PFOS-exposed testes, with key oxidative stress/spermatogenesis-related genes labeled. *H,* interaction network analysis illustrating the links between key genes involved in PFOS-induced oxidative stress, mitochondrial dysfunction, and reproductive toxicity. DEG, differentially expressed gene; PFOS, perfluorooctanesulfonic acid.

Furthermore, KEGG pathway enrichment showed substantial changes in oxidative phosphorylation, steroid hormone biosynthesis, peroxisome function, and glutathione metabolism (Fig. 2*D*), linked to mitochondrial energy, redox balance, and hormone synthesis. This suggests that PFOS may impair testicular function by disrupting these systems. In addition, hypoxia signaling (HIF-1), RNA metabolism, and stress responses were enriched, suggesting broader regulation issues. Gene set enrichment analysis further confirmed these findings, revealing significant enrichment of pathways in post-transcriptional regulation, cellular detoxification, ATP transport, and spermatogenesis (Fig. 2*E*). Critical genes, such as Trpv1, glutathione peroxidase (Gpx5), Sirt1, and Fos, were central in co-expression networks linked to oxidative stress, mitochondrial function, cell junction integrity, and reproductive processes (Fig. 2, *F*–*H*), possibly mediating PFOS-induced testicular injury. To validate the transcriptomic findings, representative genes associated with antioxidant defense, mitochondrial function, and reproductive regulation were examined independently. RT-qPCR showed that PFOS exposure (5 mg/kg/d) significantly reduced the expression of *Nfe2l2*, *Sod2*, *Gpx4*, *Ppargc1a*, *Tfam*, *Ndufs1*, *Atp5f1a*, *Stra8*, *Cyp11a1*, and *Sox9* (Fig. S1*A*). Consistently, the protein levels of Nrf2, HO-1, ATP5A, CYP11A1, STRA8, and GPX4 were decreased following PFOS exposure (Fig. S1, *B* and *C*). These results independently confirmed the disruption of antioxidant defense, mitochondrial function, and reproductive processes indicated by the transcriptomic analysis. Overall, these findings demonstrated that PFOS alters gene networks linked to oxidative stress, mitochondrial dysfunction, and spermatogenesis, with redox imbalance and mitochondrial impairment as critical factors in male reproductive toxicity.

### PFOS induces oxidative stress and mitochondrial dysfunction in GC-2 cells

Having demonstrated that redox imbalance and mitochondrial impairment are critical factors in male reproductive toxicity exposure to PFOS, we specifically investigated how PFOS induced mitochondrial dysfunction and oxidative stress. GC-2 spermatocyte cells were exposed to 10, 25, and 50 μM PFOS for 24 h (Fig. 3*A*), concentrations confirmed to be subcytotoxic by CCK-8 assay (Fig. S2) and consistent with previous studies in GC-2 cells (31). Mitochondrial superoxide levels assessed using MitoSOX staining, dose-dependently increased in mitochondrial ROS production compared to the control, with the highest levels at 50 μM (Fig. 3*B*). Intracellular ROS levels also substantially increased following PFOS exposure, approximately 6-fold at 50 μM (Fig. 3, *C* and *D*), suggesting mitochondrial oxidative damage as a critical ROS source. Mitochondrial membrane potential further confirmed cells shifting from red to green fluorescence with PFOS exposure compared to control, indicating mitochondrial membrane depolarization (Fig. 3, *E* and *F*).

**Figure 3:**
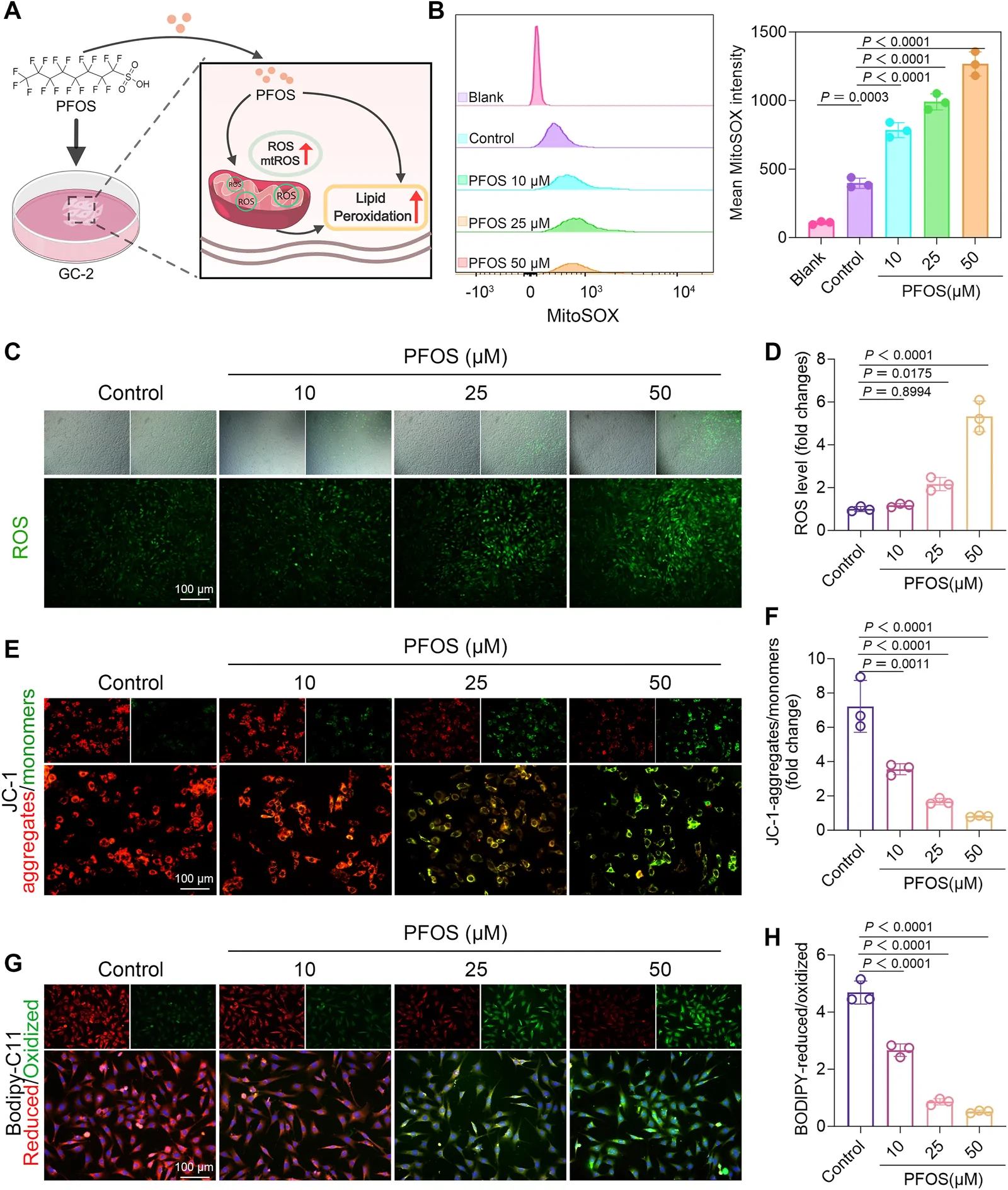
**PFOS induces mitochondrial oxidative stress and mitochondrial dysfunction in GC-2 cells.***A*, schematic illustration of PFOS treatment protocol in GC-2 cells. *B,* flow cytometric analysis of mitochondrial superoxide levels using MitoSOX staining in GC-2 cells exposed to PFOS (10, 25, and 50 μM). Histograms *(left)* and quantitative analysis of mean MitoSOX fluorescence intensity *(right)*. *C,* fluorescence images of intracellular ROS levels following PFOS exposure. The scale bar represents100 μm. *D,* quantification of intracellular ROS levels expressed as fold changes relative to control. *E,* JC-1 staining images of mitochondrial membrane potential (Δψm) in GC-2 cells following PFOS exposure. *Red* fluorescence indicates JC-1 aggregates (intact Δψm), and *green* fluorescence indicates JC-1 monomers (depolarized mitochondria). The scale bar represents 100 μm. *F,* quantification of the JC-1 aggregate-to-monomer fluorescence ratio. *G*, fluorescence images of lipid peroxidation using BODIPY-C11 staining. Reduced *(red)* and oxidized *(green)* forms are shown. The scale bar represents 100 μm. *H,* quantification of reduced-to-oxidized ratio from BODIPY-C11 fluorescence staining. Data are presented as mean ± SD, n = 3. PFOS, perfluorooctanesulfonic acid; ROS, reactive oxygen species.

Given that lipid peroxidation is a critical downstream consequence of uncontrolled ROS production and a driver of mitochondrial oxidative damage, we investigated lipid peroxidation using BODIPY-C11 staining. Results showed that 50 μM PFOS exposure triggered a gradual increase in oxidized (green) fluorescence and a decrease in (red) fluorescence (Fig. 3*G*), while quantification of the reduced-to-oxidized fluorescence ratio showed a dose-dependent decrease (Fig. 3*H*), consistently indicating increased lipid peroxidation. Malondialdehyde (MDA), a critical marker of lipid peroxidation, significantly increased in PFOS-exposed groups (Fig. S3*A*), which further confirmed lipid peroxidation product accumulation.

Furthermore, we next investigated how PFOS induced oxidative stress. Results showed that antioxidant defense capacity was markedly impaired, with the key antioxidant enzyme activities, such as superoxide dismutase (SOD), catalase (CAT), and GPx, decreased in a dose-dependent manner following PFOS exposure (Fig. S3, *B*–*D*). Collectively, these findings demonstrate that PFOS can induce excessive mitochondrial ROS production, leading to mitochondrial dysfunction, lipid peroxidation, and impairment of cellular antioxidant defenses in GC-2 cells.

### Mitochondrial ROS mediates PFOS-induced spermatocyte injury

To explore whether mitochondrial ROS causally contributes to PFOS-induced spermatocyte injury, GC-2 cells were co-treated with PFOS and the mitochondria antioxidant Mito-TEMPO (Fig. 4*A*). Flow cytometry and quantitative analysis showed that PFOS exposure markedly increased mitochondrial superoxide levels, whereas Mito-TEMPO treatment significantly reduced mitochondrial ROS production (Fig. 4*B*). Similarly, intracellular ROS levels elevated after PFOS exposure effectively decreased by Mito-TEMPO (Fig. 4, *C* and *D*), indicating mitochondrial ROS critically drives cellular oxidative stress. JC-1 staining and biochemical assays further confirmed that PFOS disrupted mitochondrial membrane potential, reduced ATP levels, and impaired complexes I, IV, and V activities, all of which were reversed by Mito-TEMPO co-treatment (Figs. 4, *F*, *G* and S4). In addition, BODIPY-C11 staining demonstrated that mitochondrial ROS inhibition markedly reduced lipid peroxidation levels (Fig. 4, *H* and *I*). Furthermore, Mito-TEMPO treatment restored antioxidant enzyme activities (SOD, CAT, and GPx) while significantly reducing MDA accumulation compared to PFOS exposure alone (Fig. 4*E*). These findings suggest that suppression of mitochondrial ROS effectively preserves mitochondrial integrity and prevents oxidative damage and collapse of cellular antioxidant defense systems.

**Figure 4:**
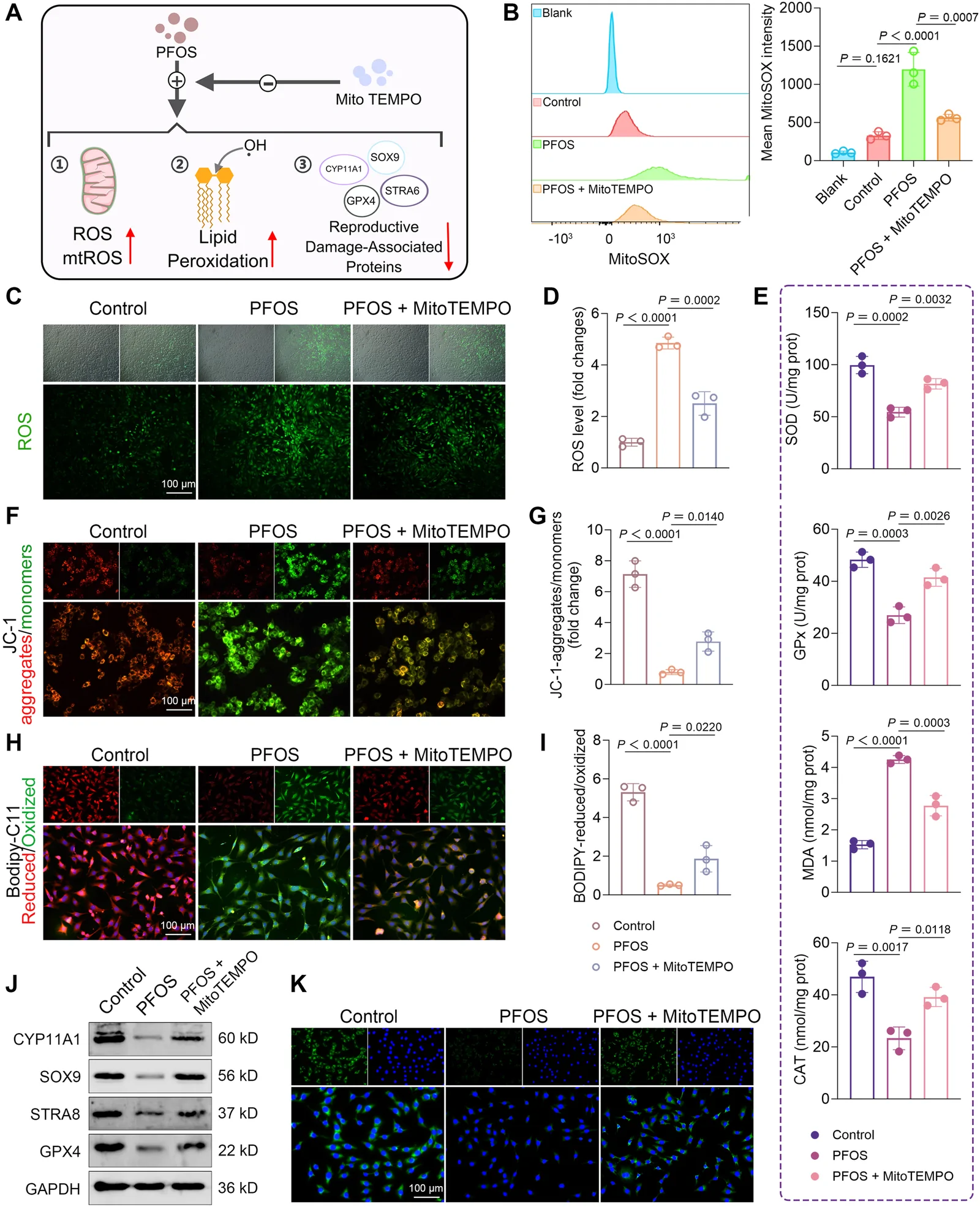
**PFOS-induced injury to spermatogenic cells depends on mitochondrial ROS production.***A,* schematic illustration of the experimental design showing PFOS exposure with or without co-treatment of MitoTEMPO in GC-2 cells. *B,* flow cytometric analysis of mitochondrial superoxide levels using MitoSOX staining. Histograms *(left)* and quantitative analysis of mean MitoSOX fluorescence intensity *(right)*. *C,* fluorescence images of intracellular ROS levels. The scale bar represents 100 μm. *D,* quantification of intracellular ROS levels expressed as fold changes relative to the control group. *E,* quantification of antioxidant enzyme markers superoxide dismutase, catalase, and GPx, and levels of MDA in GC-2 cells following PFOS exposure with or without MitoTEMPO treatment. *F,* JC-1 staining images of mitochondrial membrane potential (Δψm). *Redfluorescence* indicates JC-1 aggregates, and *greenfluorescence* indicates JC-1 monomers. The scale bar represents 100 μm. *G*, quantification of the JC-1 aggregate-to-monomer fluorescence ratio. *H,* fluorescence images of lipid peroxidation assessed by BODIPY-C11 staining. The scale bar represents100 μm. *I,* quantification of reduced -to-oxidized BODIPY-C11 fluorescence ratio. *J,* Western blot analysis of spermatogenesis-related proteins CYP11A1, SOX9, STRA8, and GPX4 in GC-2 cells. GAPDH served as a loading control. All western blots are representative of three independent experiments. *K,* immunofluorescence images of DDX4 expression in control, PFOS-treated, and PFOS + MitoTEMPO–treated cells. Nuclei were counterstained with DAPI. Data are presented as mean ± SD, n = 3. The scale bar represents 100 μm. GPX, glutathione peroxidase; PFOS, perfluorooctanesulfonic acid; ROS, reactive oxygen species.

To further investigate whether mitochondrial ROS is critical for PFOS-induced impairment of spermatogenic function, we performed Western blot analysis of key reproductive proteins. Results showed that PFOS exposure significantly downregulated the expression of key reproductive proteins including CYP11A1, SOX9, STRA8, and GPX4, whereas Mito-TEMPO co-treatment markedly restored their expression levels without affecting their basal expression when given alone (Figs. 4*J*, S5 and S6). Consistently, immunofluorescence staining revealed that PFOS-induced reduction of the germ cell marker DDX4 was significantly reversed by Mito-TEMPO (Figs. 4*K* and S7). These results demonstrate that mitochondrial ROS mediates PFOS-induced oxidative damage and spermatocyte dysfunction, and targeting it rescues mitochondrial function and spermatogenic capacity.

### PFOS regulates FTO-dependent m^6^A modification through mitochondrial ROS

Research has shown that PFOS significantly induces ROS production in various cell types (32), and under certain conditions, FTO inhibition can lower ROS levels (33). To investigate whether mitochondrial ROS links PFOS exposure to epitranscriptomic dysregulation, we assessed the m^6^A regulatory machinery in the specific GC-2 cell type. Western blot analysis showed that PFOS exposure significantly reduced the protein expression of the m^6^A demethylase FTO in a dose-dependent manner (Fig. 5, *A* and *B*). In contrast, the expression levels of m^6^A-related proteins such as METTL3, METTL14, ALKBH5, WTAP, YTHDF1, and YTHDF2 were unchanged (Fig. S8), suggesting FTO as a key target of PFOS in the m^6^A machinery system. Furthermore, PFOS-induced FTO downregulation occurred independently of cell death, as PFOS-treated cells maintained high viability without activation of inflammatory cell death pathways (Figs. S2 and S9). Consistently, dot blot analysis revealed a significant increase in global m^6^A levels following PFOS exposure (Fig. 5*C*), which was further quantified by an independent m^6^A assay showing an approximately 2.2-fold increase, partially reversed by Mito-TEMPO to approximately 1.3-fold (Fig. S10), confirming that mitochondrial ROS contributes to PFOS-induced m^6^A accumulation.

**Figure 5:**
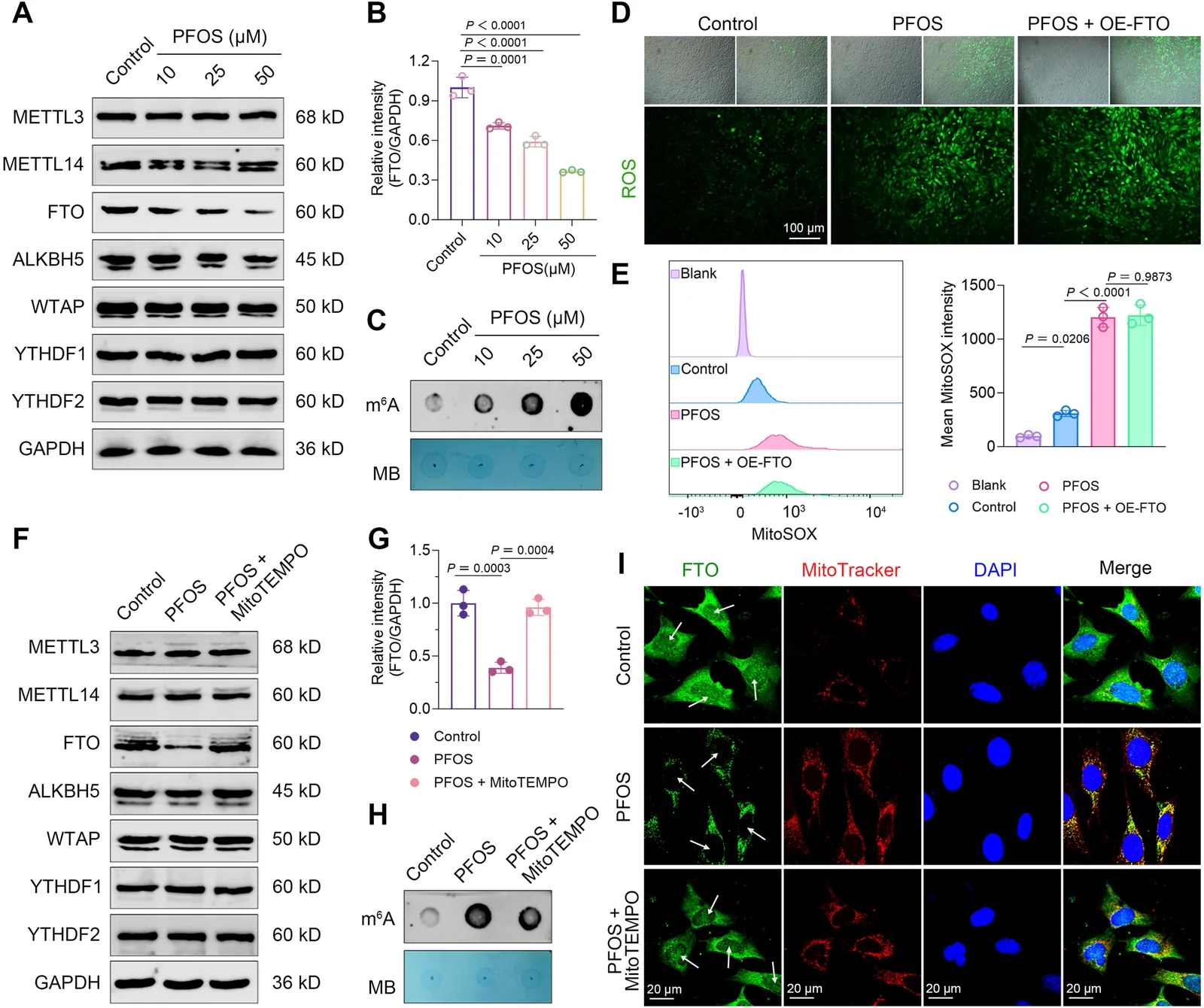
**PFOS induces m^6^A hypermethylation *via* mitochondrial ROS-mediated regulation of the FTO/m^6^A axis.***A,* Western blot analysis of m^6^A regulatory proteins (METTL3, METTL14, FTO, ALKBH5, WTAP, YTHDF1, and YTHDF2) in cells exposed to PFOS (10, 25, and 50 μM). GAPDH served as a loading control. *B,* protein quantification of FTO expression levels normalized to GAPDH. *C, dot blot* analysis of global m^6^A levels following PFOS exposure, with MB staining serving as the RNA loading control. *D,* fluorescence images of intracellular ROS levels using DCFH-DA staining in Control, PFOS, and PFOS + OE-FTO groups. The scale bar represents = 100 μm. *E,* flow cytometric analysis and quantification of mitochondrial ROS levels using MitoSOX staining. *F,* Western blot analysis of m^6^A regulatory proteins in cells treated with PFOS in the presence or absence of the mitochondrial ROS scavenger MitoTEMPO. *G,* quantification of FTO protein expression normalized to GAPDH. *H, dot blot* analysis of global m^6^A levels following PFOS and PFOS + MitoTEMPO treatment. *I*, Immunofluorescence costaining of FTO (*green*) and mitochondria (MitoTracker, *red*), with nuclei counterstained by DAPI *(blue)*. The scale bar represents = 20 μm. All western blots are representative of three independent experiments. Data are presented as mean ± SD, n = 3. MB, methylene blue; PFOS, perfluorooctanesulfonic acid; ROS, reactive oxygen species.

Although the FTO-directly-regulates-ROS concept is emerging, several recent studies have largely focused either on PFOS-treated specific cell types or on FTO functioning as an m^6^A demethylase that modulates specific genes, thereby affecting ROS levels (32, 33). To elucidate this concept, we overexpressed FTO (OE-FTO) in GC-2 cells to investigate whether FTO directly affects ROS production under PFOS exposure. Results showed that OE-FTO did not significantly affect either mitochondrial or intracellular ROS levels induced by PFOS (Figs. 5, *D*, *E* and S11), suggesting that FTO does not act upstream of ROS. Mechanistically, PFOS itself suppressed FTO primarily through accelerated protein degradation rather than reduced mRNA stability (Fig. S12), indicating that the decrease in FTO abundance is a consequence of cellular stress rather than a primary defect in FTO gene expression. In line with this, scavenging mitochondrial ROS with Mito-TEMPO markedly restored FTO protein expression and reduced global m^6^A methylation levels (Fig. 5, *F*–*H*), without affecting the expression of other m^6^A machinery regulators (Fig. S13). This indicates that mitochondrial ROS acts upstream of FTO and is required for PFOS-induced m^6^A dysregulation. Notably, FTO downregulation was not specific to PFOS, as H_2_O_2_ treatment also significantly reduced FTO protein abundance in a NAC-reversible manner (Fig. S14, *A* and *B*). PFOS suppressed FTO at both the transcriptional and post-translational levels, whereas H_2_O_2_ only reduced FTO protein without affecting *Fto* mRNA (Fig. S14*C*), indicating that PFOS exerts additional transcriptional suppression beyond general oxidative stress. Furthermore, immunofluorescence analysis showed that FTO predominantly localized in the nucleus, whereas PFOS exposure reduced nuclear FTO and increased its mitochondrial overlap, effects reversed by Mito-TEMPO (Figs. 5*I* and S15). These results indicate that m^6^A accumulation is primarily driven by total FTO loss, whereas reduced nuclear FTO may further compromise demethylation capacity. PFOS significantly reduced ATP content, complex I/IV/V activities, and mitochondrial-encoded transcripts *mt-Co1*, *mt-Nd1*, *mt-Cytb*, and *mt-Atp6*, none of which were restored by FTO overexpression (Fig. S16), indicating that the mitochondrial relocalization of FTO is a consequence rather than a driver of mitochondrial dysfunction. Collectively, these results indicate mitochondrial ROS regulates FTO expression and localization, contributing to PFOS-induced m^6^A hypermethylation.

### m^6^A epitranscriptomic profiling shows PFOS affects spermatogenesis genes

Methylated RNA immunoprecipitation sequencing (MeRIP-seq) identified 1639 hypermethylated and 1817 hypomethylated peaks in PFOS-exposed testes compared with controls, indicating that PFOS remodels the m^6^A landscape in a transcript-specific manner rather than simply increasing or decreasing m^6^A uniformly across all transcripts (Fig. 6*A*). The comparable numbers of hyper- and hypomethylated peaks do not contradict the increased global m^6^A abundance detected by dot blot and quantitative m^6^A assays, as MeRIP-seq counts each differential peak independently of its enrichment intensity, width, transcript abundance, or methylation stoichiometry, whereas global assays measure cumulative m^6^A signal across total RNA. Thus, overall m^6^A levels can increase even when the number of hypomethylated peaks equals or exceeds that of hypermethylated peaks, particularly when methylation gains occur in highly abundant transcripts or exhibit greater enrichment intensity than methylation losses. Distribution analysis of m^6^A peaks revealed that PFOS exposure altered m^6^A sites enrichment, predominantly in the coding sequence and near stop codons, with noticeable changes in the 5′ and 3′ untranslated regions (UTRs) (Fig. 6, *B* and *C*), suggesting effects of PFOS on mRNA stability and translation. Motif analysis further revealed shifts in the m^6^A sites sequence characteristics, with an increased methylation motif following PFOS exposure (Fig. 6*D*), indicating alterations in m^6^A deposition patterns and in RNA–protein interactions.

**Figure 6:**
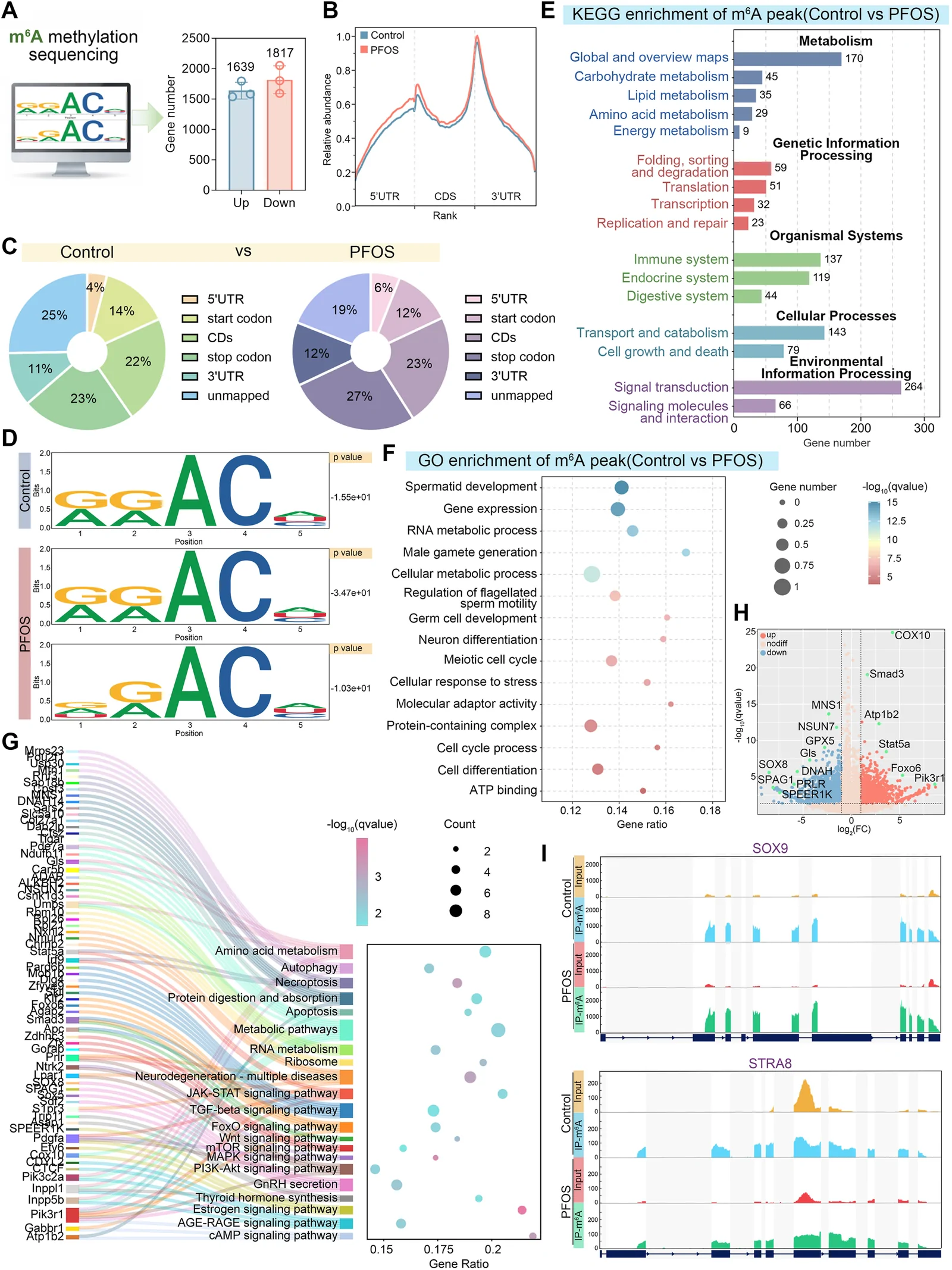
**m^6^A sequencing and epitranscriptomic analysis of PFOS-induced changes in mouse testes.***A,* m^6^A sequencing analysis of genes in PFOS-exposed. *B,* distribution of m^6^A peaks across 5′untranslated region, coding sequence, and 3′untranslated region regions in PFOS-treated and control groups. *C,* pie chart of m^6^A peaks distribution in genomic regions. *D*, motif analysis of m^6^A binding sites in PFOS-treated testes. *E,* KEGG enrichment analysis of m^6^A peaks. *F,* GO enrichment analysis of m^6^A peaks involvement in biological processes, molecular functions, and cellular components, particularly spermatogenesis pathways. *G, sankey and bubble plots* showing the pathways enriched in m^6^A peaks of PFOS-exposed testes. *H, volcano plot* showing the *top* 15 key genes linked to endocrine and reproductive functions. *I,* m^6^A peak distribution in reproductive-related target genes SOX9 and STRA8 following PFOS exposure. ROS, reactive oxygen species.

Functional enrichment analysis revealed that genes with differential m^6^A modification are significantly associated with biological processes such as spermatogenesis, steroid hormone biosynthesis, metabolic pathways, and oxidative phosphorylation (Fig. 6, *E* and *F*), which are critical in male reproductive function and cellular energy metabolism. Sankey diagram and pathway enrichment analyses confirmed that methylated transcripts are enriched in reproductive and metabolic pathways (Fig. 6*G*), suggesting coordinated epitranscriptomic regulation. Notably, reproductive genes like SOX9, STRA8, CYP11A1, and GPX4 exhibited significant m^6^A modification (Fig. 6*H*). Additionally, visualization of m^6^A peak distribution further confirmed substantial changes in m^6^A enrichment patterns following PFOS exposure (Fig. 6*I*), suggesting that PFOS may regulate gene expression through epitranscriptomic mechanisms. Collectively, these results demonstrate that PFOS exposure induces widespread remodeling of the testicular m^6^A epitranscriptome, preferentially targeting genes involved in spermatogenesis and steroidogenesis.

### FTO restoration rescues m^6^A dysregulation and reproductive gene suppression induced by PFOS

Having demonstrated that ROS regulates FTO expression, which contributes to PFOS-induced m^6^A hypermethylation, we next investigated the FTO-specific function in PFOS-induced epitranscriptomic dysregulation. The causal link between m^6^A hypermethylation and reproductive gene suppression was examined by treating PFOS-exposed cells with STM2457, a METTL3 inhibitor. STM2457 reversed PFOS-induced global m^6^A elevation and restored the mRNA and protein levels of CYP11A1, SOX9, STRA8, and GPX4 (Fig. S17), indicating that m^6^A hypermethylation functionally drives the repression of these genes. Whether FTO is responsible for this m^6^A-mediated suppression was then examined by assessing OE-FTO effects on m^6^A modification and reproductive gene expression in GC-2 cells (Fig. 7*A*). Dot blot analysis showed that PFOS exposure markedly increased global m^6^A levels, but OE-FTO significantly reduced m^6^A methylation, restoring levels to the control group (Fig. 7*B*). This suggests that OE-FTO is a critical regulator of PFOS-induced m^6^A remodeling. Western blot and protein quantification analysis confirmed that OE-FTO did not significantly affect the expression of m^6^A machinery proteins such as METTL3, METTL14, ALKBH5, WTAP, YTHDF1, and YTHDF2 (Figs. 7, *C*, *D* and S18). Conversely, FTO knockdown alone significantly reduced the mRNA and protein levels of CYP11A1, SOX9, STRA8, and GPX4 (Fig. S19), phenocopying PFOS exposure. Together, these gain- and loss-of-function results establish FTO as a functional mediator rather than a downstream marker of PFOS-induced reproductive gene suppression.

**Figure 7:**
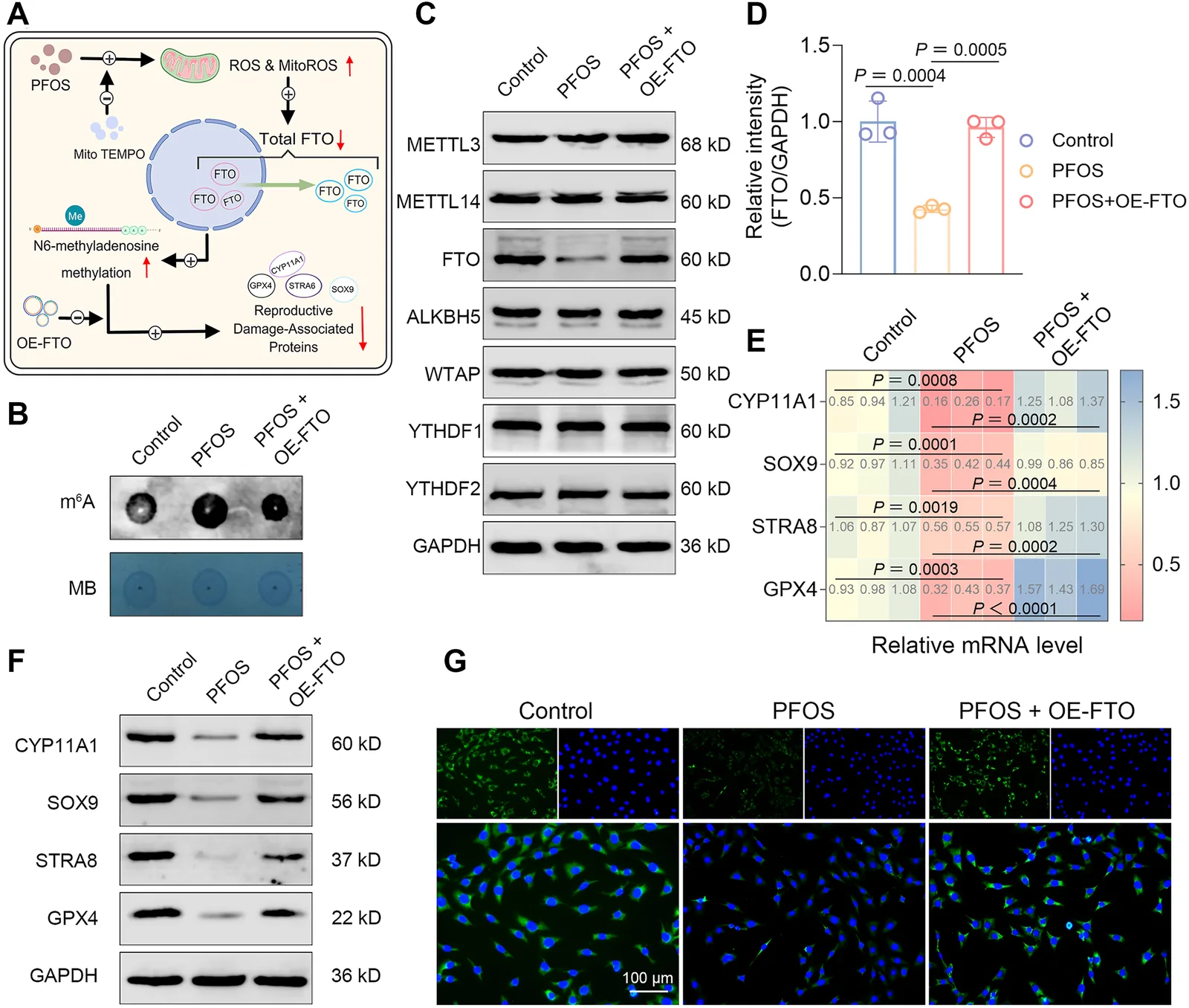
**FTO overexpression rescues PFOS-induced m^6^A hypermethylation and silencing of reproductive genes.***A,* schematic illustration of the epitranscriptomic mechanism underlying PFOS-induced germ cell dysfunction. *B, dot blot* analysis of global m^6^A RNA methylation levels in Control, PFOS, and PFOS + OE-FTO groups. MB served as an RNA loading control. *C,* western blot analysis of m^6^A machinery components (METTL3, METTL14, FTO, ALKBH5, WTAP, YTHDF1, and YTHDF2). GAPDH served as the loading control. *D,* quantification of FTO protein abundance normalized to GAPDH. *E,* RIP-qPCR analysis using anti-FTO antibodies. (*F*) Western blot analysis of spermatogenesis-related proteins CYP11A1, SOX9, STRA8, and GPX4. *G,* immunofluorescence analysis of DXX4 (*green*) with nuclear counterstaining (DAPI, *blue*). The scale bar represents = 100 μm. All western blots are representative of three independent experiments. Data are presented as mean ± SD, n = 3. GPX, glutathione peroxidase; MB, methylene blue; PFOS, perfluorooctanesulfonic acid.

To further investigate how FTO directly regulates key reproductive genes, RNA immunoprecipitation (RIP)-qPCR was performed. RIP-qPCR showed FTO antibody significantly enriched the transcripts of CYP11A1, SOX9, STRA8, and GPX4 (Fig. 7*E*), indicating that FTO binds directly to these mRNAs and may influence their stability or translation *via* m^6^A modification. Functional rescue investigation additionally showed that OE-FTO reverses PFOS-induced repression of reproductive genes. Specifically, PFOS exposure significantly reduced the protein levels of CYP11A1, SOX9, STRA8, and GPX4, whereas OE-FTO markedly restored their expression (Figs. 7*F* and S20). Furthermore, immunofluorescence analysis revealed that PFOS exposure decreased germ cell marker DDX4 expression, but OE-FTO effectively restored DDX4 levels (Figs. 7*G* and S21), indicating recovery of spermatogenic cell traits. Overall, these results indicate FTO directly regulates reproductive genes through m^6^A modification, and that restoration of FTO expression effectively rescues PFOS-induced epitranscriptomic dysregulation and spermatogenic impairment.

### Targeting mitochondrial ROS alleviates PFOS-induced testicular injury and restores FTO/m^6^A regulation *in vivo*

Encouraged by the consistent *in vitro* findings, we next validated the role of mitochondrial ROS in PFOS-induced reproductive toxicity *in vivo*, mice were concurrently co-treated with PFOS and the mitochondria-targeted antioxidant Mito-TEMPO for 8 weeks (Fig. 8*A*). Histological analysis revealed PFOS exposure significantly induced structural damage to the testis, including seminiferous tubule atrophy, expansion of interstitial space, and disruption of tubular architecture, these damages were substantially alleviated by Mito-TEMPO treatment (Fig. 8*B*). Morphometric analysis further confirmed that Mito-TEMPO significantly improved Johnsen scores, seminiferous tubule-to-interstitium ratio, and reduced interstitial expansion compared with PFOS exposure alone (Fig. 8, *F*–*I*). Functional recovery of spermatogenesis was also evidenced by increased testis weight relative to body weight, restored sperm count, and reduced sperm abnormality rates compared with the PFOS group (Fig. 8, *C*–*E*), indicating improved reproductive function. In addition, serum testosterone and luteinizing hormone (LH) levels were quantified by ELISA. PFOS reduced testosterone and elevated LH, indicating impaired steroidogenesis with compensatory HPG axis activation, both partially reversed by Mito-TEMPO (Fig. S22).

**Figure 8:**
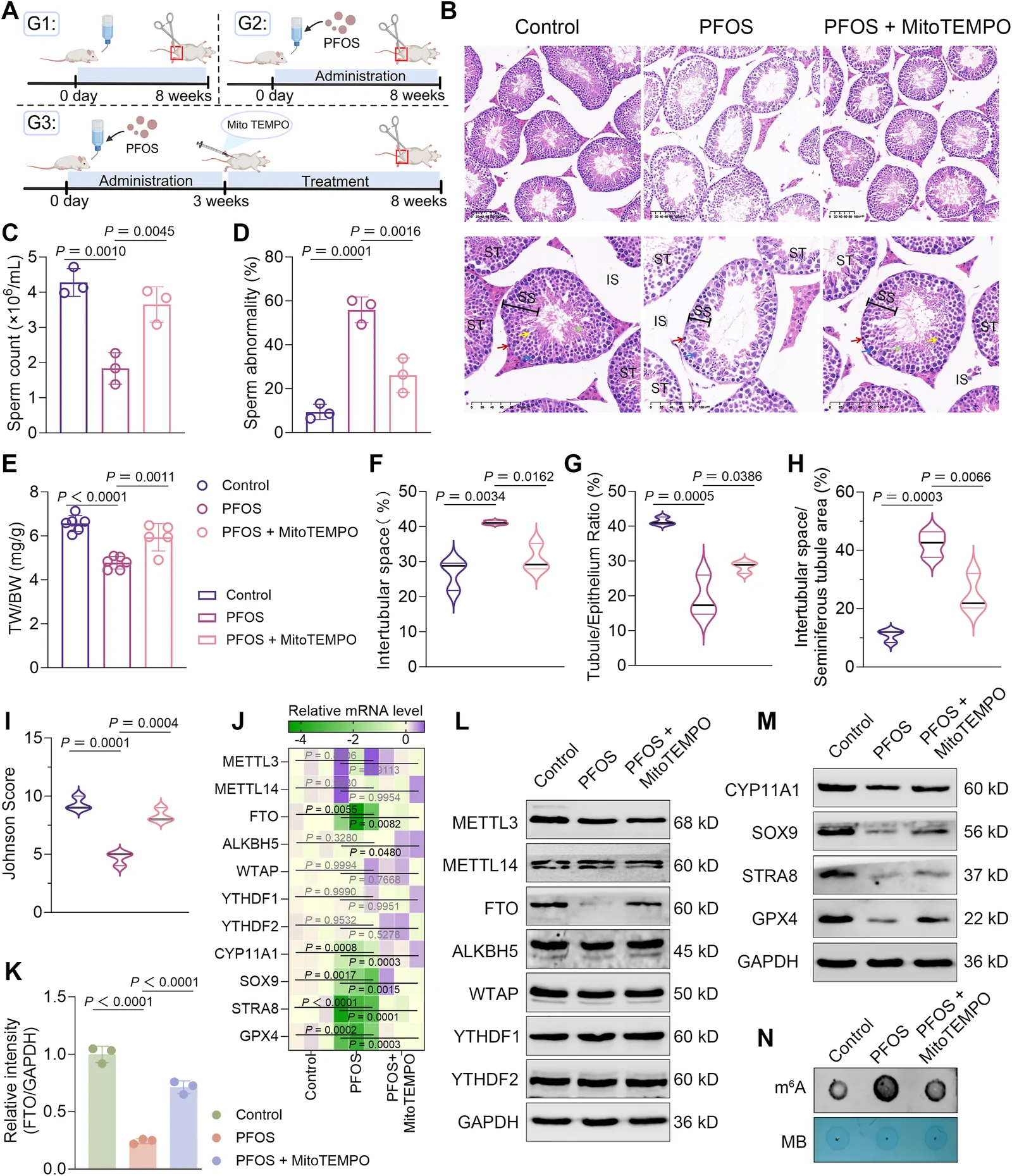
**Targeting redox-sensitive m^6^A regulation alleviates PFOS-induced testicular dysfunction *in vivo*.***A,* schematic illustration of the animal experimental design showing PFOS exposure and MitoTEMPO administration. *B,* H&E-stained sections of testicular tissue from Control, PFOS, and PFOS + MitoTEMPO groups. The scale bar represents = 100 nm. *C,* quantification of sperm count and *(D*) sperm abnormality rate. *E,* quantification of testis weight–to–body weight ratio. *F–I,* quantitative morphometric analyses of intertubular space, tubule-to-epithelium ratio, seminiferous tubule area, and Johnsen score. *J,* heatmap analysis of relative mRNA expression levels of spermatogenesis-related genes in testicular tissue. *K,* quantification of FTO protein expression normalized to GAPDH. *L,* western blot analysis of m^6^A regulatory proteins expression (METTL3, METTL14, FTO, ALKBH5, WTAP, YTHDF1, and YTHDF2). *M,* western blot analysis of spermatogenesis-related proteins CYP11A1, SOX9, STRA8, and GPX4. *N, dot blot* analysis of global m^6^A levels in testicular RNA, with MB staining as a loading control. All western blots are representative of three independent experiments. Data are presented as mean ± SD, n = 3. GPX, glutathione peroxidase; MB, methylene blue; PFOS, perfluorooctanesulfonic acid.

At the molecular level, Mito-TEMPO effectively restored redox homeostasis in the testis by recovering antioxidant enzyme activities (SOD, CAT, and GPx) and reducing MDA levels induced by PFOS exposure (Fig. S23), confirming that mitochondrial ROS critically contribute to PFOS-induced oxidative stress *in vivo*. Consistently, decreasing mitochondrial ROS reversed PFOS-induced epitranscriptomic alterations. Western blot analysis showed that PFOS exposure significantly decreased FTO expression, whereas Mito-TEMPO treatment restored FTO protein levels without significantly affecting other m^6^A regulatory proteins (Figs. 8, *J*–*L* and S24). Additionally, dot blot analysis demonstrated that Mito-TEMPO significantly reduced PFOS-induced global m^6^A hypermethylation (Fig. 8*N*), restoring epitranscriptomic balance.

The restoration of m^6^A regulation was linked to the recovery of reproductive gene expression. PFOS exposure significantly suppressed the expression of critical genes for spermatogenesis and steroidogenesis, such as CYP11A1, SOX9, STRA8, and GPX4, at both mRNA and protein levels (Figs. 8, *J*, *H* and S25). Mito-TEMPO treatment effectively rescued the expression of these genes, supporting the role of mitochondrial ROS in mediating their dysregulation. Collectively, these results demonstrate that mitochondrial ROS drives PFOS-induced testicular dysfunction *in vivo* by regulating the FTO/m^6^A axis, and that reducing mitochondrial ROS effectively restores testicular structure, function, and epitranscriptomic homeostasis.

## Discussion

PFOS, a persistent organic pollutant, has been increasingly linked to adverse effects on male reproductive health (8, 30), thereby rising male infertility rates worldwide. This study revealed that PFOS causes male reproductive failure by inducing mitochondrial ROS, which, in turn, activate FTO-dependent m^6^A hypermethylation, silencing key spermatogenic genes, leading to testicular injury and dysfunction. This framework linked PFOS to mitochondrial damage, epitranscriptomic changes, and male reproductive harm, providing new insights into PFOS male reproductive toxicity. Notably, long-term exposure to 0.1 mg/kg/d PFOS reduced the testis-to-body weight ratio and disrupted seminiferous tubule architecture, highlighting the potential reproductive risk associated with low-level environmental exposure, findings that align with previous studies (34). Structural testicular disruption and impairment of spermatogenesis are hallmark features of PFOS-induced reproductive toxicity (35), which align with our findings of seminiferous tubule atrophy, epithelial thinning, and interstitial expansion, suggesting dysfunction of Sertoli and Leydig cells. This is significant, as sertoli cell damage may cause germ cell detachment and defective spermatogenesis, whereas Leydig cell dysfunction can disrupt testosterone synthesis and the spermatogenic microenvironment (2), both of which can worsen reproductive function. Mechanistically, beyond direct testicular effects, disruption of the liver–gut microbiota–testis axis and endocrine interference through estrogen receptor activation, androgen receptor antagonism, and hypothalamic–pituitary–testicular axis suppression (30, 36, 37, 38), also contributes to PFOS toxicity.

Transcriptomic analysis showed DEGs enriched in oxidative phosphorylation and glutathione metabolism pathways, indicating mitochondrial dysfunction. Mitochondria are vital for energy, redox balance, and germ cell development during spermatogenesis (39). Research supports that PFAS compounds are mitochondrial toxicants. PFOA and PFOS impair human sperm by inducing mitochondrial-mediated apoptosis, decreasing membrane potential, and disrupting redox balance (40). Our study showed that low-dose PFOS (0.1 mg/kg/day) causes superoxide overproduction, lipid peroxidation, and reduces antioxidant levels in GC-2 spermatocytes, consistent with other research (10, 41). Mito-TEMPO, a mitochondrial antioxidant, rescues these defects, confirming that mitochondrial ROS drive PFOS-induced oxidative damage. Excessive mitochondrial ROS impair reproduction by disrupting membrane potential, ATP production, meiosis, and flagella formation (42), damaging mitochondrial DNA (43, 44), and inducing germ cell apoptosis *via* the mitochondrial permeability transition pore (45). Mitochondrial dynamics and organelle crosstalk also contribute to environmental reproductive toxicity (46, 47, 48).

This study identified a key epitranscriptomic link between mitochondrial ROS and reproductive toxicity. Notably, PFOS exposure dose-dependently suppresses the m^6^A demethylase FTO, with no effect on other m^6^A machinery components (METTL3, METTL14, ALKBH5, WTAP, YTHDF1/2). This FTO downregulation was not a consequence of cell death, as PFOS-treated cells maintained high viability without activation of inflammatory cell death pathways. Furthermore, FTO downregulation was not specific to PFOS, as H_2_O_2_ treatment also reduced FTO protein in a NAC-reversible manner. However, unlike PFOS, which suppressed FTO at both transcriptional and post-translational levels, H_2_O_2_ only affected FTO protein without altering its mRNA, suggesting that PFOS exerts additional transcriptional suppression beyond general oxidative stress. The selective targeting of FTO among m^6^A regulatory proteins is noteworthy, as previous studies linked FTO to male reproductive injuries from various environmental toxicants, but not specifically PFOS. Bisphenol A exposure during postnatal testicular development reduces FTO, raises m^6^A levels, and causes oxidative damage (49). Similarly, bisphenol F induces ferroptosis *via* FTO-mediated m^6^A regulation of FTH1 (50). Our study further showed that PFOS-induced mitochondrial ROS suppress FTO and shifts its localisation from the nucleus to the mitochondria, an effect prevented by Mito-TEMPO, indicating that mitochondrial ROS serve as a signaling hub that links environmental chemical exposure to epitranscriptomic dysregulation. Quantitative colocalization analysis confirmed that PFOS reduced nuclear FTO while increasing its mitochondrial overlap, indicating that m^6^A accumulation is primarily driven by total FTO loss, whereas the accompanying loss of nuclear FTO may further compromise demethylation capacity. Since FTO is the only affected demethylase, m^6^A hypermethylation likely results from its loss rather than an overall change in the m^6^A machinery.

Furthermore, m^6^A-seq revealed widespread changes in the testicular epitranscriptome, with hyper- and hypomethylated peaks predominantly in coding regions and near stop codons, consistent with m^6^A’s known roles in mRNA stability and translation (51). Genes with altered m^6^A were enriched in pathways critical for male reproduction, like spermatogenesis, steroid biosynthesis, and oxidative phosphorylation. Key highly methylated transcripts included CYP11A1, SOX9, STRA8, and GPX4, all crucial for germ cell development and function. RIP-qPCR confirmed FTO binds directly to these transcripts, indicating FTO prevents excessive m^6^A methylation. Unfortunately, FTO suppression by PFOS-induced mitochondrial ROS leads to hypermethylation of mRNA transcripts and decreased protein expression. Mechanistically, OE-FTO restored hypermethylation and the protein deficits, demonstrating that the FTO/m^6^A axis is essential downstream of mitochondrial ROS. Our *in vivo* results further supported this mechanism. Oral PFOS exposure in mice caused testicular damage, reduced sperm, and abnormalities, all of which were significantly alleviated by Mito-TEMPO administration. Notably, Mito-TEMPO restored FTO levels, reversed m^6^A hypermethylation, and rescued CYP11A1, SOX9, STRA8, and GPX4. These findings show the mitochondrial-ROS–FTO–m^6^A axis is active *in vivo*, and targeting mitochondrial ROS could break the cycle of oxidative and epitranscriptomic damage, suggesting mitochondria-targeted antioxidants might mitigate PFOS-related reproductive harm.

The present findings have broader implications for environmental epigenetics and epitranscriptomics. While PFAS-induced DNA methylation changes in sperm affect offspring metabolic health have been documented (28), our study shows PFOS can reprogram the testis's m^6^A epitranscriptome. Since m^6^A modification is reversible and dynamic (52), it may serve as early biomarkers or intervention targets for PFOS exposure before permanent epigenetic damage occurs. The re-localization of FTO to mitochondria suggests it has context-dependent functions beyond nuclear m^6^A demethylation. Although the molecular basis of FTO relocalization and the roles of other m^6^A regulators in redox signaling remain unclear, validation across different PFAS and human populations is still required. Further research may clarify whether mitochondrial-localized FTO influences ROS production or senses mitochondrial stress. These findings provide insight into the interplay between oxidative stress and epitranscriptomic regulation and may inform future risk assessment of PFAS exposure.

## Conclusion

In summary, this study identifies mitochondrial ROS-driven epitranscriptomic dysregulation as a central mechanism underlying PFOS-induced male reproductive toxicity (Fig. 9). Chronic exposure to environmentally relevant doses of PFOS caused dose-dependent testicular injury, seminiferous tubule disruption, and impaired spermatogenesis in mice, accompanied by marked oxidative stress and mitochondrial dysfunction. Mechanistically, PFOS increased mitochondrial ROS production, leading to mitochondrial depolarization, lipid peroxidation, and collapse of antioxidant defenses. Importantly, mitochondrial ROS acted upstream of the m^6^A demethylase FTO, suppressing its expression and promoting its relocalization from the nucleus to mitochondria, thereby inducing global m^6^A hypermethylation. Epitranscriptomic remodeling preferentially affected genes essential for spermatogenesis and steroidogenesis, while restoration of FTO expression reversed PFOS-induced m^6^A dysregulation and rescued reproductive gene expression. Notably, scavenging mitochondrial ROS with Mito-TEMPO effectively alleviated oxidative damage, restored FTO/m^6^A homeostasis, and improved reproductive outcomes both *in vitro* and *in vivo*. Together, these findings establish a mitochondrial ROS–FTO/m^6^A axis as a previously unrecognized redox-sensitive pathway in PFOS-induced reproductive injury, and highlight mitochondrial redox imbalance and epitranscriptomic disruption as potential mechanistic biomarkers and therapeutic targets for PFAS-associated male reproductive dysfunction.

**Figure 9:**
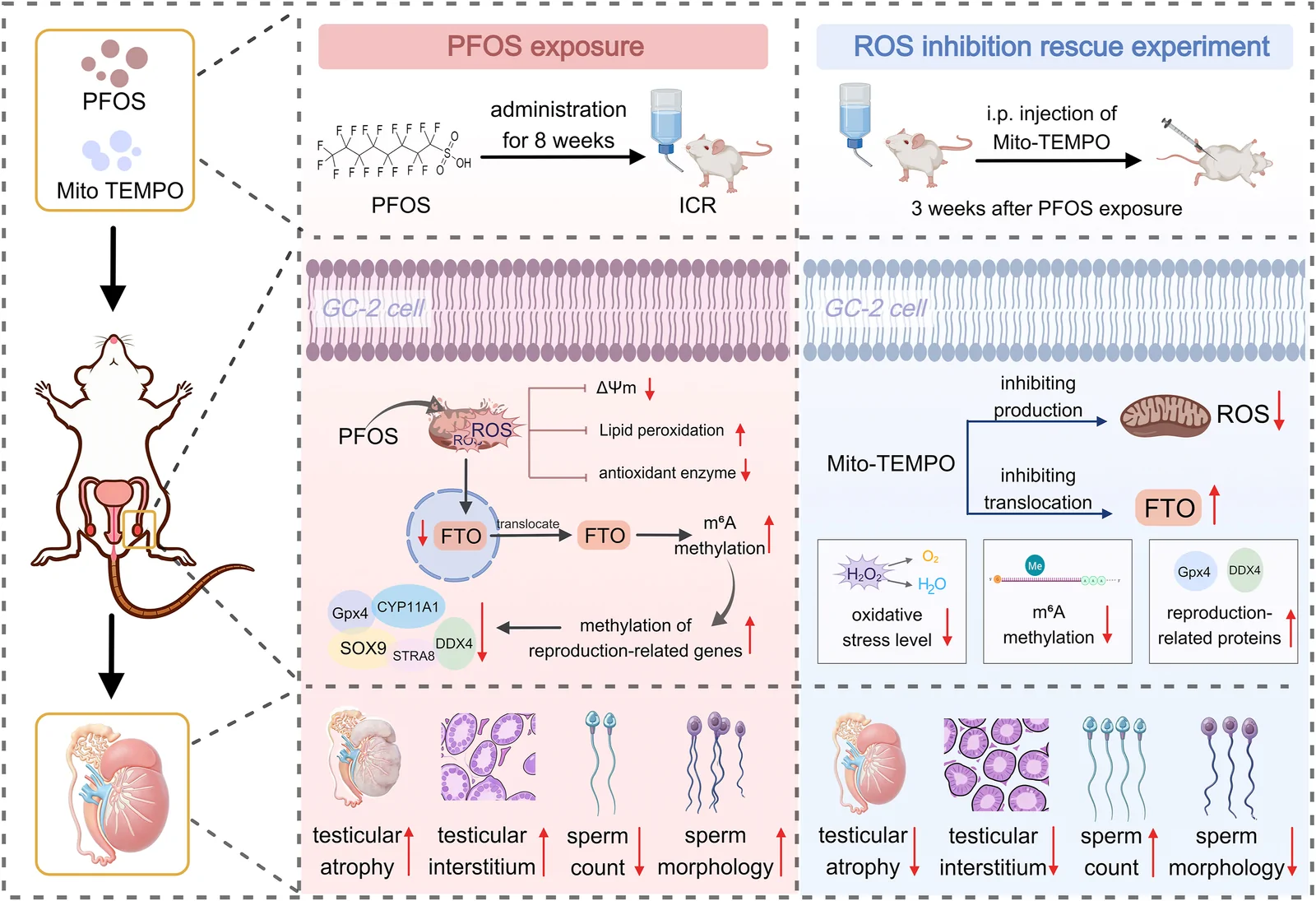
**Schematic illustration of mitochondrial ROS–FTO/m^6^A axis mediating PFOS-induced male reproductive toxicity**.

## Experimental procedures

### Reagents, materials, and bacterial strains

Perfluorooctane sulfonate (PFOS, T923214) were purchased from macklin. Mito-TEMPO (HY-112879) were purchased from MedChemExpress. The fluorescent probes MitoSOX Red (HY-D1055) and MitoTracker Deep Red FM (HY-D1783) were also obtained from MedChemExpress. 2′,7′-Dichlorofluorescin diacetate (DCFH-DA, D6470), JC-1 mitochondrial membrane potential assay kit (M8650), and assay kits for SOD, BC5165, catalase (CAT, BC0205), glutathione peroxidase (GPx, BC1195), and MDA, BC0025 were purchased from Solarbio. The fluorescent lipid peroxidation sensor C11-BODIPY581/591 (SML3717) was supplied by Sigma-Aldrich.

Primary antibodies targeting the following proteins were acquired from Proteintech: METTL3(15073-1-AP), METTL14(26158-1-AP), FTO(27226-1-AP), ALKBH5(16837-1-AP), WTAP(10200-1-AP), YTHDF1(17479-1-AP), YTHDF2(24744-1-AP), CYP11A1(13363-1-AP), SOX9(55152-1-AP), STRA8(68071-1-lg), GPX4(30388-1-AP), DDX4(30388-1-AP). Secondary antibodies, including HRP-conjugated goat anti-rabbit and goat anti-mouse, and Alexa Fluor 488-conjugated secondary antibody, were obtained from Proteintech and Huaan Biotechnology. Antibodies against glyceraldehyde-3-phosphate dehydrogenase (GAPDH, R1210-1) were sourced from HUABIO.

### Cell experiments analysis

#### Cell culture and treatment

The mouse spermatocyte-derived GC-2 cell line was purchased from Procell and cultured in DMEM-High Glucose medium (Bio-Channel) supplemented with 10% fetal bovine serum (GIBCO) and 1% penicillin-streptomycin (Solarbio, P1410) at 37 °C in a 5% CO_2_humidified incubator. For PFOS exposure, cells were treated with PFOS at concentrations of 10, 25, or 50 μM for 24 h. Control cells were treated with an equivalent volume of serum-free high-glucose medium. For mitochondrial ROS inhibition, cells were co-treated with 25 μM MitoTEMPO for 24 h. For FTO overexpression, cells were transfected with FTO overexpression plasmid or empty vector using Lipofectamine 3000 (Thermo Fisher Scientific, L3000001) according to the manufacturer's instructions.

#### Cell viability assay

GC-2 cells were seeded in 96-well plates at a density of 1 × 10^4^ cells per well and incubated overnight. Cells were then treated with various concentrations of PFOS (0, 10, 25, 50, 75, and 100 μM) for 24 h. After treatment, 10 μl of CCK-8 solution was added to each well and incubated for 1 h at 37 °C. Absorbance was measured at 450 nm using a microplate reader. Cell viability was calculated as the percentage of absorbance relative to the control group.

#### Mitochondrial superoxide detection

MitoSOX Red staining was used to detect mitochondrial superoxide levels. After treatment, cells were stained with 5 μM MitoSOX Red at 37 °C for 30 min in the dark. Following staining, cells were washed with PBS, and fluorescence intensity was measured using a flow cytometer (Olympus, excitation/emission: 510/580 nm).

#### Intracellular ROS detection

GC-2 cells were stained with 10 μM 2′,7′-dichlorofluorescin diacetate. Fluorescence intensity was measured with a fluorescence microscope (Olympus), and intracellular ROS levels were quantified using ImageJ software.

#### Mitochondrial membrane potential detection

The cationic fluorescent dye JC-1 was used to detect and analyze the mitochondrial membrane potential (ΔΨm). GC-2 cells were stained with 2 μg/ml JC-1 dye at 37 °C for 20 min in the dark. Following staining, cells were washed twice with PBS, and fluorescence images were captured using a fluorescence microscope (Olympus). Mitochondrial membrane potential was expressed as the ratio of red (JC-1 aggregates, excitation/emission: 525/590 nm) to green (JC-1 monomers, excitation/emission: 488/530 nm) fluorescence intensity, quantified using ImageJ software (National Institutes of Health, Bethesda, MD; https://imagej.net/ij/).

#### Lipid peroxidation detection

The fluorescent probe BODIPY-C11 was used to detect and quantify lipid peroxidation. GC-2 cells were stained with 2 μM BODIPY-C11 in serum-free medium at 37 °C in the dark for 30 min. Subsequently, cells were washed twice with PBS to remove unbound probes. Nuclei were counterstained with DAPI. A fluorescence microscope (Olympus) was used to assess lipid peroxidation: reduced lipids emit red fluorescence (excitation wavelength: 581 nm, emission wavelength: 591 nm), while oxidized lipids emit green fluorescence (excitation wavelength: 488 nm, emission wavelength: 510 nm). The fluorescence intensity was quantified in ImageJ, and the ratio of reduced to oxidized fluorescence was calculated to determine the level of cellular lipid peroxidation.

#### Evaluation of antioxidant activity

The activities of antioxidant enzymes and levels of lipid peroxidation were evaluated in GC-2 cells. After the indicated treatment, cells were collected and lysed in radioimmunoprecipitation assay (RIPA) lysis buffer on ice for 30 min. The lysates were centrifuged at 12,000 rpm for 15 min at 4 °C, and the supernatant was collected. Total protein concentration was determined using the bicinchoninic acid (BCA) method according to the kit instructions. Subsequently, oxidative stress-related indicators in the supernatant were detected, including MDA, SOD, CAT, and Gpx. Commercial kits were used to detect enzyme activities and metabolite levels, and the levels of each biomarker were measured strictly according to the corresponding kit instructions.

#### Measurement of ATP content and mitochondrial respiratory chain complex activities

Intracellular ATP content was measured using an ATP Assay Kit according to the manufacturer's instructions. Briefly, GC-2 cells were lysed, and the supernatant was mixed with luciferase reagent. Luminescence was measured using a luminometer. Mitochondrial respiratory chain complex I, IV, and V activities were assessed using corresponding assay kits according to the manufacturer's protocols. Activities were normalized to total protein concentration and expressed as relative values compared with the control group.

### Animals and PFOS exposure protocol

Male ICR mice (6–8 weeks old, 20–25 g) were bred in the animal center of Jiangsu Normal University (JSNU). All mice were housed in a specific pathogen-free facility under controlled temperature (22 ± 2 °C) and humidity (50 ± 10%), with a 12-h light/12-h dark cycle and free access to standard chow and sterilized water. For dose–response assessment, mice were randomly divided into four groups (n = 6 per group) and exposed to PFOS *via* drinking water at doses of 0 (control, water), 0.1 (ultra-low-dose), 0.5 (low-dose), and 5 (medium-dose) mg·kg^−1^ day^−1^ for 8 weeks. For mitochondrial ROS intervention, mice were randomly divided into three groups (n = 6 per group): control (water), PFOS (5 mg kg^−1^ day^−1^), and PFOS (5 mg kg^−1^ day^−1^) with Mito-TEMPO (1 mg kg^−1^ day^−1^, intraperitoneal injection). PFOS was administered *via* drinking water for 8 weeks, and Mito-TEMPO was injected daily. PFOS solutions were freshly prepared each week based on average daily water consumption and body weight. All animal experiments were approved by the Institutional Animal Care and Use Committee of Jiangsu Normal University (Approval No. [JSNU-IACUC-2026005]) and conducted in accordance with relevant guidelines and regulations.

### Histological and morphometric analysis

Testicular tissues were fixed in 4% paraformaldehyde for 48 h, embedded in paraffin, sectioned into 5 μm thick slices, and stained with H&E. Histopathological changes were observed under an optical microscope. Seminiferous tubule area, interstitial space, and tubule-to-epithelium ratio were quantified using ImageJ software (NIH). Spermatogenesis maturity was assessed using the Johnsen score (range 1–10).

### Sperm count and morphological analysis

After euthanasia, bilateral cauda epididymides were isolated and placed in pre-warmed PBS. Tissues were minced and incubated at 37 °C for 10 min to allow spermatozoa to swim out. Sperm count was determined using a hemocytometer. For morphological analysis, sperm smears were stained with eosin–aniline black and examined under an optical microscope at 1000× magnification. Sperm morphology was assessed according to the criteria established by the World Health Organization laboratory manual, with at least 200 sperm examined per sample. A morphologically normal spermatozoon was defined as having a smooth, oval-shaped head of appropriate dimensions, a well-defined acrosome covering 40% to 70% of the head surface, a slender midpiece, and a tail without sharp bends or irregularities. Sperm deviating from these morphological features, including head defects such as amorphous, tapered, round, or double heads; midpiece abnormalities such as bent neck, cytoplasmic droplets, or irregular thickness; and tail defects such as coiled, short, broken, or multiple tails, were classified as abnormal. The percentage of morphologically abnormal sperm was calculated for each sample.

### Hormone measurement

Blood samples were collected from mice by retro-orbital bleeding at the end of the 8-weeks exposure period. Serum was separated by centrifugation at 3000 rpm for 15 min at 4 °C and stored at −80 °C until analysis. Serum testosterone and LH levels were measured using commercial ELISA kit according to the manufacturer's instructions. Absorbance was read at 450 nm using a microplate reader, and hormone concentrations were calculated from standard curves.

### RNA extraction and transcriptome analysis

Total RNA was extracted from testicular tissues using TRIzol reagent (CW Bio, CW0580S) according to the manufacturer’s instructions. RNA purity and concentration were measured using a NanoDrop 2000 spectrophotometer (Thermo Fisher Scientific), and RNA integrity was evaluated prior to subsequent library preparation. RNA libraries were constructed using a poly(A) mRNA enrichment strategy, followed by RNA fragmentation, first- and second-strand cDNA synthesis, end repair, adapter ligation, and PCR amplification. The qualified libraries were subjected to high-throughput sequencing on an Illumina platform. Raw sequencing reads were filtered to remove adapter sequences and low-quality reads, generating clean reads for downstream analysis. Clean reads were aligned to the reference genome using a standard alignment algorithm, and gene expression levels were quantified based on normalized read counts. Differential expression analysis was performed using the DESeq2 package, with screening criteria set as |log2 fold change| > 1 and adjusted *p* value < 0.05. GO and KEGG enrichment analyses were conducted to identify significantly enriched biological processes and signaling pathways associated with differentially expressed genes.

### m^6^A sequencing analysis

Total RNA was extracted from testicular tissues of control and PFOS-exposed mice (5 mg kg^−1^ day^−1^, n = 3 per group) using TRIzol reagent according to the manufacturer’s instructions. RNA purity and concentration were measured using a NanoDrop 2000 spectrophotometer (Thermo Fisher Scientific), and RNA integrity was assessed using an Agilent 2100 Bioanalyzer. MeRIP-seq was performed using a standard protocol. Briefly, fragmented RNA was incubated with an anti-m^6^A antibody for immunoprecipitation, followed by purification of m^6^A-enriched RNA fragments. Both input and immunoprecipitated RNA samples were used for library construction, including cDNA synthesis, adapter ligation, and PCR amplification. The resulting libraries were subjected to high-throughput sequencing on an Illumina platform. Raw sequencing reads were processed to remove adapter sequences and low-quality reads. Clean reads were aligned to the reference genome using a standard alignment algorithm. m^6^A peaks were identified based on enrichment in immunoprecipitated samples relative to input controls using a peak-calling algorithm, with thresholds set at fold enrichment ≥ 2 and *p* < 0.05. Motif analysis, peak distribution across transcript regions (5′UTR, coding sequence, and 3′UTR), and functional enrichment analyses, including GO and KEGG, were performed to characterize the biological significance of m^6^A-modified transcripts.

### Evaluation of antioxidant activity in testicular tissue

The activities of antioxidant enzymes and levels of lipid peroxidation were evaluated in testicular tissues. After the 8-weeks exposure period, testicular tissues were collected from control, PFOS-exposed, and PFOS with MitoTEMPO groups. Tissue samples were homogenized in RIPA lysis buffer on ice, and the homogenates were centrifuged at 12,000 rpm for 15 min at 4 °C. The supernatant was collected, and total protein concentration was determined using the BCA method according to the kit instructions. Subsequently, oxidative stress-related indicators in the supernatant were detected, including MDA, SOD, CAT, and GPx. Commercial kits were used to perform specific detection of enzyme activities and metabolite levels, and the levels of each biomarker were measured strictly following the corresponding kit instructions.

### Western blotting

Total protein was extracted from GC-2 cells using RIPA buffer (CW Bio, CW2333s) containing protease inhibitors (Beyotime, ST506). Proteins were separated by 12% SDS-PAGE, transferred to PVDF membranes (GVS), and blocked with 5% non-fat milk. Membranes were incubated overnight at 4 °C with primary antibodies against METTL3, METTL14, FTO, ALKBH5, WTAP, YTHDF1, YTHDF2, CYP11A1, SOX9, STRA8, GPX4, and GAPDH, followed by HRP-conjugated goat anti-rabbit secondary antibodyor HRP-conjugated goat anti-mouse secondary antibody. Bands were visualized using ECL reagent (Biosharp) and imaged with a chemiluminescence documentation system (Tanon). GAPDH expression served as the loading control for normalization, and quantitative densitometric analysis was performed using ImageJ.

### RNA stability and protein stability assays

For detection of FTO mRNA expression under PFOS stimulation, GC-2 cells were treated with gradient concentrations of PFOS (0, 10, 25, 50 μM) for 24 h, total RNA was isolated and subjected to RT-qPCR to quantify relative FTO transcript levels. For mRNA stability assay, control and PFOS-pretreated GC-2 cells were incubated with 5 μg/ml actinomycin D to inhibit transcription, and cells were harvested at 0, 2, 4, 6, 8 h to detect residual FTO mRNA *via* RT-qPCR, normalized to the 0 h value of each group. To measure FTO protein stability, cycloheximide (20 μg/ml) was added to block new protein synthesis in vehicle or PFOS-exposed cells, cell lysates were collected at sequential time points (0–8 h), and Western blot was used to quantify remaining FTO protein abundance.

### m^6^A dot bot assay

Total RNA (500 ng/μl) was spotted onto a nylon membrane (Amersha), cross-linked under UV (254 nm, 1200 mJ/cm^2^), and blocked with 5% non-fat milk. The membrane was incubated with anti-m^6^A antibody (1:1000, Proteintech) overnight at 4 °C, followed by HRP-conjugated secondary antibody. Signals were visualized using ECL. Methylene blue staining served as the loading control.

### Immunofluorescence staining and co-localization analysis

#### Immunofluorescence staining

GC-2 cells were fixed with 4% paraformaldehyde, permeabilized with 0.1% Triton X-100, and blocked with 5% BSA. Cells were incubated with an anti-DDX4 primary antibody overnight at 4 °C, followed by a fluorescent secondary antibody. Nuclei were counterstained with DAPI. Images were captured using an Olympus fluorescence microscope and quantified using ImageJ.

#### Mitochondrial colocalization analysis

GC-2 cells were stained with MitoTracker Deep Red FM to label mitochondria, fixed with methanol, permeabilized, and blocked. Cells were incubated with anti-FTO primary antibody overnight at 4 °C, followed by Alexa Fluor 488-conjugated secondary antibody. Nuclei were counterstained with DAPI. Images were captured using a confocal laser scanning microscope (Leica) and analyzed for colocalization (Pearson's correlation coefficient) using ImageJ.

### Quantitative real-time PCR (RT-qPCR)

cDNA was synthesized from total RNA using a reverse transcription kit. RT-qPCR was performed using SYBR Green Master Mix on a QuantStudio 5 Real-Time PCR system with the following cycling conditions: 95 °C for 30 s, followed by 40 cycles of 95 °C for 5 s and 60 °C for 30 s. Relative gene expression was calculated using the 2ˆ–ΔΔCt method and normalized to Gapdh. The primers used for RT-qPCR are listed in Table S1.

### RIP-qPCR

RIP was performed using a Pure Binding RIP kit (Geneseed), according to the manufacturer's protocol.GC-2 cell lysates were incubated with magnetic beads coupled with anti-FTO (5 μg, Proteintech) antibody and control rabbit IgG (Abcam) at 4 °C overnight. RT-qPCR was performed to quantify the transcripts of reproduction-related genes in the immunoprecipitated RNA and in total RNA from whole-cell lysates. The primers used for RT-qPCR are listed in Table S2.

### FTO overexpression and knockdown

For FTO overexpression, GC-2 cells were transfected with FTO overexpression plasmid or empty vector using Lipofectamine 3000 according to the manufacturer's instructions. For FTO knockdown, cells were transfected with FTO-targeting siRNA (si-FTO) or negative-control siRNA (si-NC) using Lipofectamine 3000. After 48 h of transfection, cells were harvested for RNA extraction or protein lysis. Knockdown efficiency was confirmed by RT-qPCR and Western blot.

### STM2457 treatment and quantitative m^6^A assay

GC-2 cells were treated with PFOS (50 μM) in the presence or absence of STM2457 (10 μM) for 24 h. Global m^6^A levels were measured using an m^6^A RNA Methylation Quantification Kit according to the manufacturer's instructions. Briefly, total RNA was extracted and incubated with capture antibody, followed by detection antibody, and absorbance was measured at 450 nm. The m^6^A levels were normalized to the input RNA concentration and expressed as relative values compared with the control group.

### Statistical analysis

Data are presented as mean ± standard deviation from at least three independent experiments. Statistical analysis was performed using GraphPad Prism 10 software (GraphPad Software, Boston, MA). Multiple group comparisons were conducted using one-way ANOVA followed by Tukey's *post hoc* test. A *p*-value < 0.05 was considered statistically significant.

## Data availability

Data will be made available on request.

## Supporting information

This article contains supporting information.

## Conflict of interest

The authors declare that they have no conflicts of interest with the contents of this article.
