# Supplementary material for: PFOS-induced mitochondrial ROS impairs male reproductive function by suppressing FTO-dependent m6A RNA demethylation
Source: J Biol Chem. 2026 Aug 26;302(10):113488. doi: 10.1016/j.jbc.2026.113488 (PMC13629210; doi:10.1016/j.jbc.2026.113488)
Supplement: Supplementary file 1 [file mmc1.docx]

Supporting Information

**PFOS-induced mitochondrial ROS impairs male reproductive function by suppressing FTO-dependent m⁶A RNA demethylation**

Yipeng Pang^1#^, Shiqing Yang^1#^, Yuenan Wang^1^, Fructueux Modeste Amona^1^, Rong Zhou^1^, Zilu Liu^1^, Chuanbei Tian^1^*, Xi Chen^1^*

^1^ Institute of Cellular and Molecular Biology, School of Life Science, Jiangsu Normal University, Xuzhou, 221116, Jiangsu, China;

^#^These authors contributed equally.

***Correspondence:**

**Xi Chen**

Email: [cxvirus@jsnu.edu.cn](mailto:cxvirus@jsnu.edu.cn)

**Chuanbei Tian**

E-mail: chuanbei_tian@jsnu.edu.cn

**Supplementary tables**

**Table S1. Primers used for RT-qPCR in this study.**

| Primer name | Primer sequence (5’ to 3’) |
| --- | --- |
| Gapdh-F | CCCTTAAGAGGGATGCTGCC |
| Gapdh-R | GTTCACACCGACCTTCACCA |
| Fto-F | GTGTTTTGGCTGGCTCACAG |
| Fto-R | GTCGCCATCGTCTGAGTCAT |
| Nfe2l2-F | TCTCCTAGTTCTCCGCTGCT |
| Nfe2l2-R | AACTTGTACCGCCTCGTCTG |
| Sod2-F | GTAGGGCCTGTCCGATGATG |
| Sod2-R | CGCTACTGAGAAAGGTGCCA |
| Gpx4-F | GGAGCCCATTCCTGAACCTT |
| Gpx4-R | GGTGACGATGCACACGAAAC |
| Ppargc1a-F | GCGAGAGAATTTCAGCTGCC |
| Ppargc1a-R | AGATCTGGGCAAAGAGGCTG |
| Tfam-F | CACGAAGCTCAGTAGGCAGC |
| Tfam-R | CCTCCACAGGGCTGCAATTT |
| Ndufs1-F | GGTTTTGAGGGGTCAGGGAG |
| Ndufs1-R | CCAGTCAAGGGCAAGAGGAG |
| Atp5f1a-F | CATTTTGTGCCAGTCGTCCG |
| Atp5f1a-R | ACAGCATCCTTGCAGCTTCT |
| Stra8-F | TTCCTGCGTGTTCCACAAGT |
| Stra8-R | ACTGGGTTGGTTGCCTTCTC |
| Cyp11a1-F | CGGTTACAGGATCGCCCTTT |
| Cyp11a1-R | CTCCTGCGCATAGAGAGAGC |
| Sox9-F | GTGCAAGCTGGCAAAGTTGA |
| Sox9-R | TGCTCAGTTCACCGATGTCC |
| mt-Co1-F | ACCCAATCAAACGCCTAGCA |
| mt-Co1-R | AGGACTGGAATGCTGGTTGG |
| mt-Nd1-F | ACCCAATCAAACGCCTAGCA |
| mt-Nd1-R | AGGACTGGAATGCTGGTTGG |
| mt-Cytb-F | ACCCAATCAAACGCCTAGCA |
| mt-Cytb-R | AGGACTGGAATGCTGGTTGG |
| mt-Atp6-F | ACCCAATCAAACGCCTAGCA |
| mt-Atp6-R | AGGACTGGAATGCTGGTTGG |

**Table S2. Primers used for RT-qPCR analysis of RIP-qPCR**

| Primer name | Primer sequence (5’ to 3’) |
| --- | --- |
| Cyp11a1-F | AGATCCCTTCCCCTGGTGACAATG |
| Cyp11a1-R | CGCATGAGAGTATCGACGCATC |
| Sox9-F | GACAAGCGGAGGCCGAA |
| Sox9-R | CCAGCTTGCACGTCGGTT |
| Stra8-F | GGCAGACTCTCTCTGAGGAGAAAA |
| Stra8-R | CTCTGGTTCCTGGTTTAATGGAGT |
| Gpx4-F | CTGGCAGGCACCATGTGTGCATCC |
| Gpx4-R | AGGCAGCCAGGGTGAAGCTCGAGC |

**Supplementary figures**

**Figure S1. Validation of PFOS-induced changes in genes associated with antioxidant defense, mitochondrial function, and spermatogenesis.** (A) RT-qPCR analysis of genes involved in antioxidant defense (Nfe2l2, Sod2, and Gpx4), mitochondrial biogenesis and oxidative phosphorylation (Ppargc1a, Tfam, Ndufs1, and Atp5f1a), and spermatogenesis or steroidogenesis (Stra8, Cyp11a1, and Sox9) in testicular tissues. (B-C) Representative western blots (B) and quantification (C) of Nrf2, HO-1, ATP5A, CYP11A1, STRA8, and GPX4. Data are presented as mean ± SD (n = 3).

**Figure S2. Effects of PFOS on GC-2 cell viability.** GC-2 cells were treated with increasing concentrations of PFOS (0, 10, 25, 50, 75, and 100 μM) for 24 h, and cell viability was assessed using the CCK-8 assay. Data are presented as mean ± SD (n = 3).

**Figure S3. PFOS disrupts antioxidant defense and enhances lipid peroxidation in GC-2 cells.** (A) Malondialdehyde (MDA), (B) Superoxide dismutase (SOD), (C) Catalase (CAT), and (D) Glutathione peroxidase (GPx) levels in GC-2 cells treated with PFOS (10, 25, and 50 μM). Data are presented as mean ± SD, n=3.

**Figure S4. Effects of PFOS and Mito-TEMPO on mitochondrial bioenergetic function in GC-2 cells.** (A) Relative intracellular ATP content. (B-D) Relative activities of mitochondrial respiratory chain complexes I, IV, and V, respectively. Values were normalized to the control group. Data are presented as mean ± SD (n = 3).

**Figure S5. Densitometric analysis of reproductive function–related proteins in GC-2 cells**. (A-D) Relative protein expression levels of (A) CYP11A1, (B) SOX9, (C) STRA8, and (D) GPX4 normalized to GAPDH in Control, PFOS, and PFOS + MitoTEMPO–treated GC-2 cells. Data are presented as mean ± SD, n=3.

**Figure S6. Effects of Mito-TEMPO alone on reproductive-function-related protein expression in GC-2 cells.** (A) Representative western blots of CYP11A1, SOX9, STRA8, and GPX4, with GAPDH as the loading control. (B) Corresponding densitometric quantification normalized to GAPDH. Data are presented as mean ± SD (n = 3).

**Figure S7. Quantification of immunofluorescence intensity in GC-2 cells.** Mean fluorescence intensity (MFI) of DDX4 in GC-2 cells from Control, PFOS, and PFOS + MitoTEMPO groups. Data are presented as mean ± SD, n=3.

**Figure S8. Quantitative analysis of m⁶A regulatory protein expression following PFOS exposure.** (A-F) Relative protein expression levels of METTL3, METTL14, ALKBH5, WTAP, YTHDF1, and YTHDF2 normalized to GAPDH, corresponding to the Western blot data shown in Fig. 5A. Data are presented as mean ± SD, n=3. Exact P values are shown in the graphs.

**Figure S9. Immunofluorescence co-staining of FTO and IL-1β in GC-2 cells.** (A-C): Representative images (A) and quantitative analysis of FTO (B) and IL-1β (C) fluorescence intensity. Scale bar = 50 μm. Data are presented as mean ± SD (n = 3).

**Figure S10. Quantitative analysis of global m⁶A abundance following Mito-TEMPO treatment.** Global m⁶A abundance in total RNA was measured using a quantitative m⁶A RNA methylation assay and normalized to the control group. Data are presented as mean ± SD (n = 3).

**Figure S11. Quantification of PFOS-induced intracellular ROS levels.** ROS expression levels quantification from fluorescence intensity measurements corresponding to the DCFH-DA staining shown in Fig. 5D-E. Data are presented as mean ± SD, n=3.

**Figure S12. Effects of PFOS on FTO mRNA and FTO protein stability in GC-2 cells.** (A) RT-qPCR analysis of **FTO** mRNA expression following treatment with increasing concentrations of PFOS. (B) **FTO** mRNA decay in control and PFOS-treated cells following inhibition of transcription with actinomycin D. Transcript abundance was normalized to the corresponding 0-h value. (C) CHX-chase analysis of FTO protein stability in control and PFOS-treated cells. Data are presented as mean ± SD (n = 3).

**Figure S13. Effects of mitochondrial ROS inhibition on m⁶A regulatory proteins other than FTO**. (A-F) Protein quantification of METTL3, METTL14, ALKBH5, WTAP, YTHDF1, and YTHDF2 protein levels normalized to GAPDH in Control, PFOS, and PFOS + MitoTEMPO groups, corresponding to the Western blot data shown in Fig. 4F. Data are presented as mean ± SD, n=3.

**Figure S14. Effects of H₂O₂-induced oxidative stress on FTO expression in GC-2 cells.** (A) Representative western blot of FTO. (B) Quantification of FTO protein abundance normalized to GAPDH. (C) RT-qPCR analysis of Fto mRNA expression. Data are presented as mean ± SD (n = 3).

**Figure S15. Colocalization and fluorescence intensity profile analysis corresponding to Figure 5I.** Line-scan fluorescence intensity profiles of MitoTracker (red), FTO (green), and DAPI (blue) were obtained from the indicated regions of representative GC-2 cells in the control (A), PFOS (B), and PFOS plus Mito-TEMPO (C) groups. Coincident FTO and MitoTracker intensity peaks indicate spatial colocalization of FTO with mitochondria, whereas overlap between FTO and DAPI signals indicates nuclear FTO localization.

**Figure S16. Effects of FTO overexpression on mitochondrial bioenergetics and mitochondrial transcript levels in PFOS-treated GC-2 cells.** (A) Relative intracellular ATP content. (B-D) Relative activities of mitochondrial respiratory chain complexes I, IV, and V, respectively. (E-H) RT-qPCR analysis of the mitochondrial-encoded transcripts mt-Co1, mt-Nd1, mt-Cytb, and mt-Atp6, respectively. Values were normalized to the control group. Data are presented as mean ± SD (n = 3).

**Figure S17. Effects of STM2457 on PFOS-induced m⁶A accumulation and reproductive-function-related gene expression in GC-2 cells.** (A) Global RNA m⁶A levels measured using an m⁶A RNA methylation quantification assay. (B-E) RT-qPCR analysis of Cyp11a1, Sox9, Stra8, and Gpx4 expression, respectively. (F, G) Representative western blots (F) and quantification (G) of CYP11A1, SOX9, STRA8, and GPX4, with GAPDH as the loading control. Data are presented as mean ± SD (n = 3).

**Figure S18. Quantification of m⁶A machinery components under PFOS exposure and FTO restoration.** (A-F) Protein quantification of METTL3, METTL14, ALKBH5, WTAP, YTHDF1, and YTHDF2 expression levels normalized to GAPDH in Control, PFOS, and PFOS + OE-FTO groups. Data are presented as mean ± SD, n=3.

**Figure S19. Effects of FTO knockdown on reproductive-function-related gene expression in GC-2 cells.** (A) RT-qPCR validation of FTO knockdown efficiency. (B) RT-qPCR analysis of Stra8, Gpx4, Cyp11a1, and Sox9 expression. (C-D) Representative western blots (C) and quantification (D) of CYP11A1, SOX9, STRA8, and GPX4. Data are presented as mean ± SD (n = 3).

**Figure S20. Quantitative analysis of spermatogenesis-related marker restoration following FTO overexpression.** Protein quantification of CYP11A1 (A), SOX9 (B), STRA8 (C), and GPX4 (D) expression levels normalized to GAPDH in Control, PFOS, and PFOS + OE-FTO groups

**Figure S21. Quantification of immunofluorescence signal intensity for spermatogenesis-related marker DDX4.** Mean fluorescence intensity (MFI) analysis of immunofluorescence staining signals corresponding to spermatogenesis-related marker DDX4 in Control, PFOS, and PFOS + OE-FTO groups. Data are presented as mean ± SD, n=3.

**Figure S22. Effects of PFOS and Mito-TEMPO on circulating reproductive hormone levels in mice.** (A, B) Serum testosterone (A) and LH levels (B) were quantified by ELISA. Data are presented as mean ± SD (n = 3).

**Figure S23. Effects of mitochondrial ROS inhibition on testicular oxidative stress parameters.** Activities of antioxidant enzymes (SOD, CAT, and GPx) and levels of lipid peroxidation marker MDA in testicular tissue from Control, PFOS, and PFOS + MitoTEMPO groups. Data are presented as mean ± SD, n=3.

**Figure S24. Quantitative analysis of m⁶A regulatory protein expression in mouse testes.** Densitometric quantification of METTL3, METTL14, ALKBH5, WTAP, YTHDF1, and YTHDF2 protein levels normalized to GAPDH, corresponding to Fig. 6L. Data are presented as mean ± SD, n=3.

**Figure S25. Quantitative analysis of spermatogenesis-related protein expression.** Densitometric quantification of CYP11A1, SOX9, STRA8, and GPX4 protein levels normalized to GAPDH, corresponding to Fig. 6M. Data are presented as mean ± SD, n=3.
